# Applications of Long Short-Term Memory (LSTM) Networks in Polymeric Sciences: A Review

**DOI:** 10.3390/polym16182607

**Published:** 2024-09-14

**Authors:** Ivan Malashin, Vadim Tynchenko, Andrei Gantimurov, Vladimir Nelyub, Aleksei Borodulin

**Affiliations:** 1 Artificial Intelligence Technology Scientific and Education Center, Bauman Moscow State Technical University, Moscow 105005, Russia; 2 Scientific Department, Far Eastern Federal University, Vladivostok 690922, Russia

**Keywords:** LSTM, polymer science, predictive analytics, polymer properties

## Abstract

This review explores the application of Long Short-Term Memory (LSTM) networks, a specialized type of recurrent neural network (RNN), in the field of polymeric sciences. LSTM networks have shown notable effectiveness in modeling sequential data and predicting time-series outcomes, which are essential for understanding complex molecular structures and dynamic processes in polymers. This review delves into the use of LSTM models for predicting polymer properties, monitoring polymerization processes, and evaluating the degradation and mechanical performance of polymers. Additionally, it addresses the challenges related to data availability and interpretability. Through various case studies and comparative analyses, the review demonstrates the effectiveness of LSTM networks in different polymer science applications. Future directions are also discussed, with an emphasis on real-time applications and the need for interdisciplinary collaboration. The goal of this review is to connect advanced machine learning (ML) techniques with polymer science, thereby promoting innovation and improving predictive capabilities in the field.

## 1. Introduction

### 1.1. Purpose of the Review

The convergence of machine learning (ML) and material science [1,2,3] has opened new avenues for research and application. This review aims to explore the integration of Long Short-Term Memory (LSTM) networks in polymeric sciences, focusing on their application in predicting and modeling polymer properties and processes.

The number of research articles that discuss the application of LSTM networks [4,5] in the field of polymers has seen an increase over recent years. Initially, the intersection of these two fields was relatively unexplored, but with the growing interest in applying ML to material sciences, more studies have been published. The earliest relevant publications started to appear around 2020, when LSTM networks began gaining popularity for their ability to handle sequential data, which is important in modeling time-series and dynamic processes in polymer science. Since then, the number of articles has grown steadily each year, with noticeable increases around 2022–2024 as more researchers began exploring advanced ML techniques, including LSTM, to predict polymer properties, monitor processes, and assess performance. Based on Mendeley data, there are currently 44 articles [6] with the terms “LSTM” and “polymers” in the title or abstract, highlighting the growing interest in the intersection of these fields.

The structure of this review is as follows: Section 1.2 provides a detailed description of the LSTM architecture. Section 2 explores the applications of LSTM models to polymers. Section 3 discusses the challenges associated with data acquisition and the interpretability of models. Section 4 outlines potential directions for future research. Finally, Section 5 presents the conclusions.

### 1.2. LSTM Overview

LSTM networks were introduced by Sepp Hochreiter and Jürgen Schmidhuber in 1997 [7] as a solution to the limitations of traditional RNNs [8], specifically the vanishing and exploding gradient problems that arise during backpropagation through time [9] (BPTT). Their unique architecture allows them to retain and utilize information over extended periods, making them suitable for time-series prediction and modeling dynamic systems. These issues make it difficult for standard RNNs to learn and retain long-term dependencies, an essential aspect for tasks involving sequential data.

**Figure 1:**
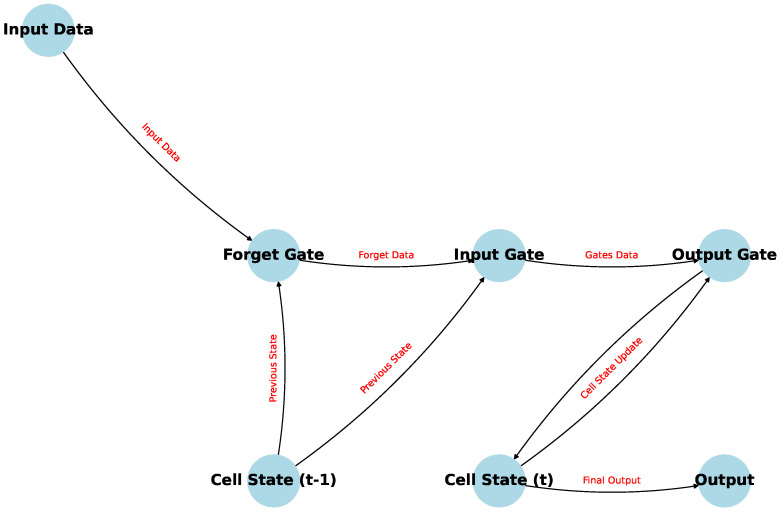
LSTM architecture diagram.

The core idea behind LSTM is the introduction of memory cells [10], which can maintain their state over time, and a gating mechanism to control the flow of information. This structure allows LSTM models to mitigate the gradient issues by ensuring that gradients can propagate more effectively over long sequences. An architecture diagram is shown in Figure 1.

Mathematically, consider a traditional RNN, where the hidden state $h_{t}$ at time step *t* is given by

(1) $$h_{t}=\tanh\left(W_{h}h_{t-1}+W_{x}x_{t}+b\right)$$

During backpropagation, the gradient $\frac{𝜕L}{𝜕h_{t}}$, where *L* is the loss function, is computed [11]. For long sequences, the recursive multiplication of gradients leads to either vanishing (values close to zero) or exploding (values diverging to infinity) gradients, making training unstable and inefficient.

LSTM overcomes this by introducing the cell state [12] $C_{t}$, which acts as a conveyor belt, allowing gradients to flow without significant alteration. The cell state is updated as follows:

(2) $$C_{t}=f_{t}\cdot C_{t-1}+i_{t}\cdot {\tilde{C}}_{t}$$

Here, $f_{t}$, $i_{t}$, and ${\tilde{C}}_{t}$ are the forget gate, input gate, and candidate cell state, respectively, as defined earlier. The forget gate $f_{t}$ determines what fraction of the previous cell state $C_{t-1}$ should be retained, while the input gate $i_{t}$ controls how much of the new candidate state ${\tilde{C}}_{t}$ should be added. This selective updating mechanism allows the LSTM to preserve relevant information across long sequences while gradually forgetting less important details [13].

The output of the LSTM unit, or the hidden state $h_{t}$, is then computed as

(3) $$h_{t}=o_{t}\cdot \tanh\left(C_{t}\right)$$

where $o_{t}$ is the output gate, which controls how much of the cell state’s information should be passed on to the next layer or time step.

This architecture enables LSTM networks to effectively learn long-term dependencies, making them particularly useful in fields such as material science, where processes and phenomena often evolve over extended periods [14,15].

An LSTM unit is composed of a cell, an input gate, an output gate, and a forget gate. These components work together to manage the flow of information through the network, allowing it to retain important features over long sequences [16,17]. This capability has impacts in material science, where processes such as stress–strain relationships [18], phase transitions [19], or diffusion phenomena [20] evolve over time. Each component plays a specific role:

- The cell state acts as the memory of the LSTM unit [21], carrying information across time steps [22]. It can retain information over long periods, enabling the network to remember past data for future predictions. The cell state is updated based on the interactions between the gates, allowing it to accumulate or forget information as needed [23].
- The input gate controls how much of the new information [24] (i.e., the candidate cell state) should be added to the cell state. This gate decides what portion of the incoming data at the current time step *t*, combined with the previous hidden state $h_{t-1}$, should be considered and stored in the cell [25]. Mathematically, it is defined as
  (4) $$i_{t}=\sigma \left(W_{i}\cdot \left[h_{t-1},x_{t}\right]+b_{i}\right)$$
  where σ is the sigmoid function, $W_{i}$ represents the weight matrix, $h_{t-1}$ is the previous hidden state, $x_{t}$ is the current input, and $b_{i}$ is the bias.
- The forget gate [26] determines how much of the previous cell state $C_{t-1}$ should be retained in the current cell state $C_{t}$. This gate is crucial for deciding which information is no longer relevant and can be “forgotten.” The forget gate’s operation is given by
  (5) $$f_{t}=\sigma \left(W_{f}\cdot \left[h_{t-1},x_{t}\right]+b_{f}\right)$$
  A value of $f_{t}$ close to 0 means that the corresponding information in the cell state will be mostly discarded, while a value close to 1 means the information will be largely retained [27].
- The output gate [28] controls what information from the cell state should be passed on to the next time step or used as the output of the current LSTM unit. It decides what part of the cell state’s information contributes to the hidden state $h_{t}$, which in turn influences the network’s predictions [29]. The output gate is calculated as
  (6) $$o_{t}=\sigma \left(W_{o}\cdot \left[h_{t-1},x_{t}\right]+b_{o}\right)$$
  The final hidden state [30] $h_{t}$ is then computed by combining the output gate’s result with the cell state, passed through a nonlinearity:
  (7) $$h_{t}=o_{t}\cdot \tanh\left(C_{t}\right)$$

Together, these gates allow the LSTM network to selectively update, retain, and discard information, making it particularly powerful for modeling complex, time-dependent processes in material science [15,31,32], such as predicting the behavior of materials under stress or modeling the progression of phase changes over time.

LSTM networks are particularly suited for predicting the stress–strain behavior of materials under various loading conditions [33,34,35]. Given a sequence of applied stresses σ(t) over time, LSTM models can predict the resulting strain ϵ(t), capturing both the immediate response and long-term effects such as creep and relaxation. The network essentially learns a mapping:

(8) ϵ(t)=LSTM(σ(t),σ(t−1),…,σ(0))

where ϵ(t) is the predicted strain at time *t*, and σ(t) represents the stress history up to that point.

In materials undergoing phase transitions [36,37], the prediction of the material’s state over time as temperature, pressure, or other conditions change is critical. LSTM can model the evolution of the phase fractions $\phi_{i}\left(t\right)$ for different phases *i* as a function of time-dependent parameters like temperature T(t):

(9) $$\phi_{i}\left(t\right)=\mathrm{LSTM}\left(T\left(t\right),T\left(t-1\right),\ldots ,T\left(0\right)\right)$$

This allows for the accurate prediction of phase compositions over a thermal cycle.

The time-dependent diffusion [38,39] of atoms or molecules in a material is another area where LSTM excels. The concentration c(x,t) of a diffusing species at position *x* and time *t* can be predicted using LSTM by training on sequences of concentration profiles:

(10) c(x,t)=LSTM(c(x,t−1),c(x,t−2),…)

This is useful in materials processing applications such as doping in semiconductors [40] or alloying in metals [41].

### 1.3. Variants of LSTM Networks

**Figure 2:**
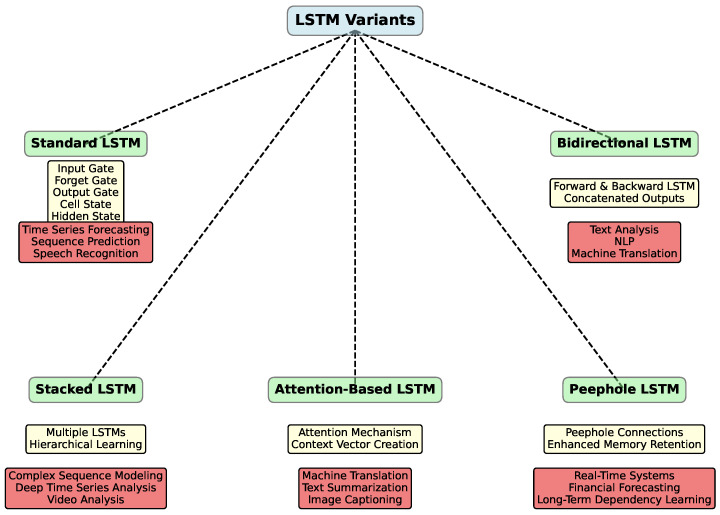
Conceptual diagram of LSTM variants.

In addition to the standard LSTM, there are several other variations of this architecture, each optimized for specific tasks and types of data. Figure 2 illustrates various LSTM network types.

For instance, Bidirectional LSTM (BiLSTM) [42] processes sequences in both directions—forward and backward—allowing the model to consider both preceding and subsequent context. Stacked LSTM [43] is a multilayered architecture where the output of one LSTM layer serves as the input for the next, helping to capture more complex patterns in the data. Peephole LSTM [44] adds direct connections between the cell state and the gates, enabling the gates to better control the information stored in the cell. Finally, Attention-Based LSTM [45] incorporates an attention mechanism, allowing the model to focus on important parts of the sequence when making predictions. These extensions of the classical LSTM make it more flexible and effective for a wide range of tasks, including time-series analysis, natural language processing, and many other applications.

#### 1.3.1. Bidirectional LSTM

Bidirectional LSTM (BiLSTM) processes input sequences in both forward and backward directions. This allows the network to have information from both past and future contexts.

- Forward LSTM: processes the sequence in the original order [46].
  (11) $$\vec{h_{t}}=\mathrm{LSTM}\left(x_{t},\vec{h_{t-1}}\right)$$
- Backward LSTM: processes the sequence in reverse order [47].
  (12) $$\overset{\leftarrow }{h_{t}}=\mathrm{LSTM}\left(x_{t},\overset{\leftarrow }{h_{t+1}}\right)$$
- Final output: concatenates the forward and backward hidden states.
  (13) $$h_{t}=\left[\vec{h_{t}},\overset{\leftarrow }{h_{t}}\right]$$

Among the advantages of this model is its ability to process the input sequence in both forward and backward directions. This bidirectional processing allows the network to capture contextual information from both past and future time steps, significantly enhancing the model’s ability to understand and predict sequential data [48]. Additionally, by leveraging information from both directions, Bidirectional LSTM models often achieve higher accuracy and better performance in tasks such as sequence labeling, speech recognition, and natural language processing [49].

However, there are also disadvantages to consider. One notable drawback is the increased computational load. The bidirectional processing doubles the computational requirements, as the model needs to process the input sequence twice [50]. This can be a limitation in real-time applications or when computational resources are constrained. Another challenge is the complexity in real-time applications. In scenarios where future data are not available, the backward pass of the Bidirectional LSTM may not be feasible, limiting its applicability [51].

#### 1.3.2. Stacked LSTM

Stacked LSTM networks involve multiple LSTM layers where the output of one LSTM layer serves as the input to the next [52]. This allows the network to capture more complex patterns in the data.

- Layer 1 LSTM [53]: processes the input sequence.
  (14) $$h_{t}^{\left(1\right)}={\mathrm{LSTM}}^{\left(1\right)}\left(x_{t},h_{t-1}^{\left(1\right)}\right)$$
- Layer 2 LSTM [54]: takes the output of Layer 1 as input.
  (15) $$h_{t}^{\left(2\right)}={\mathrm{LSTM}}^{\left(2\right)}\left(h_{t}^{\left(1\right)},h_{t-1}^{\left(2\right)}\right)$$
- Final output [55]: can be taken from the last layer’s hidden state.

Stacked LSTM models consist of multiple layers of LSTM cells, enabling the network to learn more complex patterns and hierarchical representations in the data. This increased depth improves the model’s ability to capture intricate temporal dependencies [56]. By stacking multiple LSTM layers, the model can achieve higher accuracy and better generalization on complex tasks, such as time-series forecasting and sequence classification [52].

Disadvantages of Stacked LSTM models include increased computational complexity [57]. The additional layers in a Stacked LSTM model increase the computational requirements, making it more resource-intensive to train and deploy. With more layers, there is a higher risk of overfitting [58], especially if the dataset is not sufficiently large or diverse. Regularization techniques, such as dropout, are often necessary to mitigate this issue.

#### 1.3.3. Peephole LSTM

Peephole LSTM models are a variation where the gates are connected not only to the previous hidden state $h_{t-1}$ and the input $x_{t}$ but also directly [16,59] to the cell state $C_{t-1}$. This allows the gates to have a view of the cell state, potentially improving performance.

- Peephole forget gate:
  (16) $$f_{t}=\sigma \left(W_{f}\cdot \left[h_{t-1},x_{t}\right]+V_{f}\cdot C_{t-1}+b_{f}\right)$$
- Peephole input gate:
  (17) $$i_{t}=\sigma \left(W_{i}\cdot \left[h_{t-1},x_{t}\right]+V_{i}\cdot C_{t-1}+b_{i}\right)$$
- Peephole output gate:
  (18) $$o_{t}=\sigma \left(W_{o}\cdot \left[h_{t-1},x_{t}\right]+V_{o}\cdot C_{t}+b_{o}\right)$$

Here, $V_{f}$, $V_{i}$, and $V_{o}$ are additional weight matrices associated with the cell state.

Advantages of Peephole LSTM models include additional connections, called peepholes, that allow the cell state to directly influence the gates. This design enhances the model’s ability to retain and utilize long-term dependencies, leading to improved performance in tasks that require long-term memory. Peephole connections can help stabilize the gradient flow during training, making the model more robust and easier to train [60].

Disadvantages of Peephole LSTM models include the increase in complexity of the model that the addition of peepholes causes, requiring more parameters and potentially longer training times [61]. Implementing and tuning the peephole connections can be more complex compared to standard LSTM models, requiring careful consideration of the model architecture and hyperparameters [62].

#### 1.3.4. Attention-Based LSTM

Attention mechanisms can be integrated with LSTM networks to focus on specific parts of the input sequence when making predictions [63,64]. The attention mechanism assigns a weight $\alpha_{t}$ to each time step in the input sequence.

- Attention weights [65]:
  (19) $$\alpha_{t}=\mathrm{softmax}\left(e_{t}\right)$$
  where
  (20) $$e_{t}=v^{T}\tanh\left(W_{h}h_{t}+W_{s}s_{t-1}+b_{e}\right)$$
- Context vector [66]:
  (21) $$c_{t}=\sum_{t^{\prime }}\alpha_{t^{\prime }}h_{t^{\prime }}$$
- Final output: combines the context vector with the LSTM output.
  (22) $$\tilde{h_{t}}=\tanh\left(W_{c}\left[c_{t};h_{t}\right]\right)$$

Here, $e_{t}$ is an alignment score, $W_{h}$, $W_{s}$, and $W_{c}$ are weight matrices [67,68], vs. is a vector, and $b_{e}$ is a bias term.

LSTM networks, in their various forms, offer powerful tools for sequence modeling, each variation tailored to different types of sequence data and tasks [69]. From standard LSTM to more complex architectures like Bidirectional, Stacked, Peephole, and Attention-Based LSTM models, these models are equipped to handle a wide range of challenges in time-series prediction, natural language processing, and beyond.

Advantages of Attention-Based LSTM models incorporate an attention mechanism that allows the network to focus on the most relevant parts of the input sequence [63]. This selective attention can improve the model’s performance by prioritizing important information and ignoring irrelevant data. The attention mechanism provides insights into which parts of the input sequence are most influential in the model’s predictions, making the model more interpretable and transparent [70].

A disadvantage of the attention mechanism is that it increases the complexity of the model, requiring more computational resources and potentially longer training times [71]. Implementing and tuning the attention mechanism can be challenging, as it involves additional hyperparameters and architectural considerations.

## 2. Applications of LSTM in Polymeric Sciences

**Figure 3:**
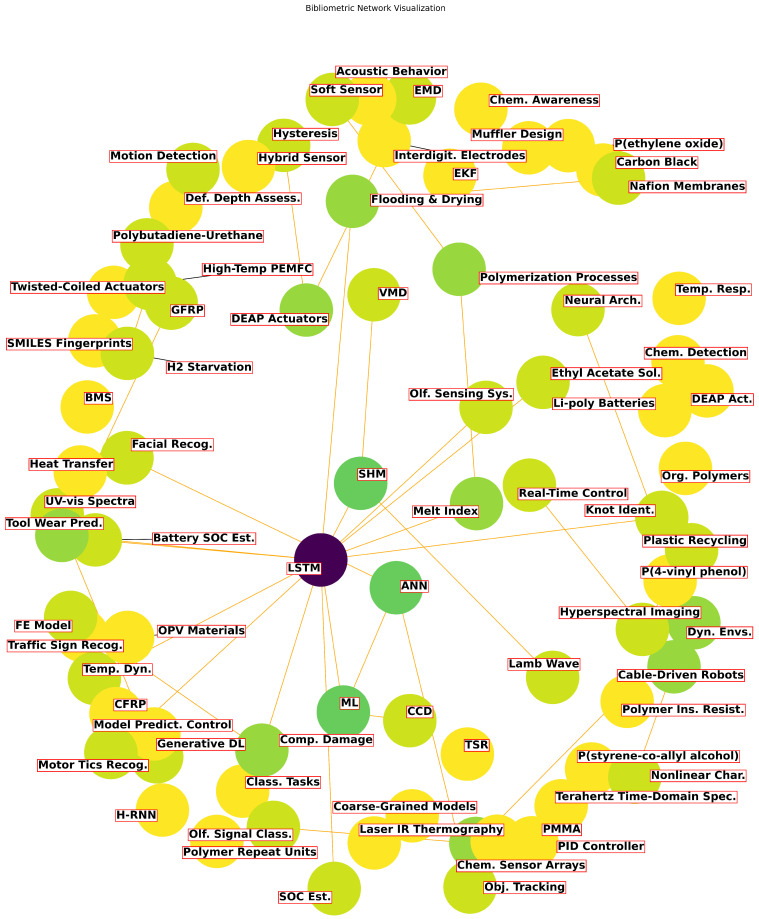
Workflow of the experimental and analytical approach used in this study.

LSTM networks have emerged as a powerful tool in the field of polymeric sciences, offering advancements in predictive modeling and sensor technologies. These networks excel in handling sequential data and time-series predictions [72,73], which is crucial for applications such as predicting polymer aging, optimizing manufacturing processes, and detecting faults in polymer composites. The bibliometric network visualization of LSTM applications in polymeric sciences, presented in Figure 3, illustrates the extensive and growing integration of LSTM networks across various polymer-related studies, highlighting their impact and versatility in enhancing the performance and reliability of polymer materials.

The word cloud uses varying shades of color to represent the frequency of word usage, with darker colors indicating more frequent mentions in the literature. For example, terms like “LSTM”, “ML”, “SHM”, and “ANN” appear in darker shades. The circles are grouped based on the strength of the relationship between the terms, with closer grouping indicating a stronger interrelationship. Terms like “Tool Wear Pred.” and “Battery SOC Est.” are closely grouped, reflecting their interconnectedness in studies that apply LSTM to monitor and predict the degradation of polymer composites. The word cloud helps to identify the most relevant and frequently discussed topics in the field, providing a visual representation of key areas where LSTM has been successfully integrated into polymer science. It also highlights emerging trends and areas of focus, guiding researchers towards potential avenues for further exploration and innovation. By examining the word cloud, we are able to quickly grasp the themes and the interrelationships between different aspects of LSTM integration in polymer science. This visual aid enhances the understanding of the current state of research and potential future directions in this interdisciplinary field.

### 2.1. Tim- Series Analysis in Polymer Systems

ML innovations for Charge-Coupled Device [74] (CCD) chips have enabled capabilities like facial recognition [75] and object tracking [76] by efficiently processing large volumes of temporal data. However, despite progress in creating chemical sensor arrays that mimic mammalian olfactory systems [77,78], limited research has been conducted into their temporal responses and the neural architectures needed for chemical awareness in dynamic environments.

To address this gap, Ryman et al. [79] developed sensors using a blend of carbon black and various organic polymers, including poly(4-vinyl phenol) [80], poly(styrene-co-allyl alcohol) [81], and poly(ethylene oxide) [82]. These sensors, when applied to interdigitated electrodes, allowed for precise resistance measurements and effective chemical detection. At the same time, LSTM networks have demonstrated exceptional performance in classification tasks, often surpassing human capabilities in areas like traffic sign recognition [83]. LSTM networks are particularly adept at managing temporal dependencies, selectively storing and forgetting states, and scaling across multiple categories, making them ideal for addressing the challenges of olfactory signal classification and processing the temporal dynamics of sensor data [84]. The integration of LSTM networks with organic polymer-based sensors is advancing olfactory sensing systems similar to how these technologies have revolutionized machine vision.

In the realm of energy systems, accurate estimation of battery state of charge [85] (SOC) remains challenging due to its nonlinearity and influence from various factors. While the extended Kalman filter [86] (EKF) is commonly used for SOC estimation, its accuracy can be compromised by uncertainties in battery models and varying conditions. Shin et al. [87] proposed a method that enhances EKF accuracy by compensating errors with an LSTM network. This approach involves training the LSTM on EKF errors and applying calibration values based on battery conditions and load profiles. The multi-LSTM structure, utilizing ensemble averaging, achieves SOC estimation with a root mean square error of less than 1%, closely matching the SOC calculated by coulomb counting, and allows for online prediction once the model is trained.

Similarly, Andrews et al. [88] evaluated three recurrent neural network architectures—ERNN [89], LSTM, and GRU [90]—for predicting the energetics of an ethyl acetate solution with a polymer–lipid aggregate [91]. Trained on extensive molecular dynamics simulation data, these models effectively reproduce time-series data but struggle with accurate short- and long-term forecasts. An in silico protocol was proposed, utilizing time patterns from the data to improve forecasts, enhancing predictions by providing a range of values consistent with energy fluctuations. This approach offers useful estimates for evaluating the necessity of long simulations in materials design.

Wang et al. [92] presented a hybrid sensor for motor tic recognition [93], integrating piezoelectric and triboelectric designs. The sensor, combining a triboelectric nanogenerator made from bionic PDMS and a piezoelectric nanogenerator using layered porous PVDF-TrFE nanofibers [94], shows an improvement in voltage output, reaching nearly 5 V. A self-powered tic recognition system utilizing a deep learning (DL) model, specifically LSTM, achieves an 88.1% recognition rate for motor tics, aiding doctors in monitoring Tourette syndrome patients [95].

In the context of fuel cell technology, degradation due to hydrogen (H2) starvation limits the lifespan of high-temperature polymer electrolyte membrane fuel cells (HT-PEM FC). Yezerska et al. [96] utilized an LSTM neural network trained on electrochemical data from $H_{2}$ starvation/regeneration experiments to predict $H_{2}$ starvation effects [97]. Simulations showed critical resistances at specific voltages, recommending a safe operational voltage range to avoid severe degradation.

Proton Exchange Membrane Fuel Cells (PEMFCs) [98], favored for green transportation, suffer from radical-induced degradation in Nafion^®^ membranes [99], leading to performance and stability issues. Benhaddouch et al. [100] introduced fluoride emission as a diagnostic model using fluoride-sensitive membranes [101] (LaF_3_/CaF_2_) in inline microsensor arrays for real-time monitoring. These sensors, coupled with LSTM algorithms, achieve high sensitivity and accuracy, providing a complementary approach for predicting PEMFC end of life [102] (EOL) and enhancing current diagnostic techniques.

In material science, Xu et al. [103] presented a method for classifying substances within glass fiber-reinforced polymer (GFRP) honeycomb structures using terahertz time-domain spectroscopy (THz-TDS). An improved one-dimensional convolutional neural network (1D-CNN) [104] model was developed and compared with LSTM and standard 1D-CNN models. The results show that the LSTM model excels with time-domain signals, while the improved 1D-CNN model is superior with frequency-domain signals.

Song et al. [105] introduced an LSTM-based soft sensor model for predicting melt index (MI) [106] in polymerization processes, which have an influence on determining polymer quality. Due to the lack of online MI measurement, traditional models struggle with the nonlinearity and complex temporal correlations of chemical processes. The LSTM model was applied to an industrial styrene–acrylonitrile (SAN) polymerization process [107], outperforming other models in prediction accuracy.

Furthermore, Song et al. [108] introduced the Self-constructed Strategy-based Reinforcement LSTM (SCRLA) [108] for predicting the nonlinear performance degradation of fiber-reinforced polymers [109] (FRP). SCRLA enhances model generalization by integrating Bayesian algorithms for hyperparameter optimization and reinforcing the learning process. This approach demonstrated superior prediction accuracy, especially with experimental data, offering an effective framework for analyzing and predicting the sequential performance of composite materials.

Finally, Goswami et al. [110] addressed the challenge of accurately measuring Glass Transition Temperature [111] ($T_{g}$) in polymers. They proposed using an LSTM model based on the Simplified Molecular-Input Line-Entry System (SMILES) structure of polymers to predict $T_{g}$. The study evaluated the model’s performance and its practical applications, offering a potentially efficient alternative to conventional methods.

As a result, LSTM networks have transformed the analysis and prediction of complex time-dependent behaviors in polymer systems. These models excel at handling the temporal dependencies inherent in these systems, offering improvements in accuracy and efficiency over traditional methods. Table 1 offers a concise overview of key articles that highlight the application of LSTM and related models in the time-series analysis of polymer systems.

### 2.2. Diagnostics and Monitoring of Polymer Materials

Recent advancements in polymer and battery technology [112] have been enhanced by DL and ML techniques. For instance, Kim et al. [113] developed a DL-based prediagnosis system for PEMFCs, using LSTM and CNN [114] combined with a bagging ensemble method [115]. By analyzing experimental time-series data from full-scale single-cell tests, this system achieves detection rates of 98.52% for flooding and 95.36% for drying, thereby improving PEMFC stability and operation.

In the field of underwater electroacoustic sensors, Ramachandran et al. [116] focused on predicting the end of life of these sensors by analyzing the degradation of their water-proof polymer insulation due to water ingress [117]. They employed LSTM networks to model and predict the degradation pattern based on measured insulation resistance [118]. This method allows for maintenance or replacement decisions without disassembling the sensors, verifying the accuracy of the predictions against actual end-of-life measurements.

Similarly, in the realm of polymer matrix composites (PMCs), Lee et al. [119] addressed the challenge of predicting tensile behavior by utilizing feature engineering combined with ML. They used Principal Component Analysis [120] (PCA) and Recursive Feature Elimination with Cross Validation [121] (RFECV) to identify the optimal features for predicting the tensile stress–strain curve [122] from test data. LSTM and Feedforward Neural Network [123] (FNN) models trained on this feature set achieved a predictive accuracy of $R^{2}=92\%$, facilitating accurate stress–strain curve predictions and simplifying PMC design.

Chistyakova et al. [124] evaluated predictive models for key quality indicators in polymer film materials [125]. They compared Adaptive Boosting of Decision Trees (AdaBoost) [126] with LSTM to predict defects such as the number of black dots per square meter. Performance was assessed using precision, recall, and $F_{1}$-score to determine the most effective model based on production data characteristics.

In the context of glass fiber-reinforced polymers [127] (GFRPs) used in marine infrastructure, Zhang et al. [128] developed an optimized ML model to predict tensile strength retention [129] (TSR) in alkaline environments. They trained seven different ML models, including LSTM and Extreme Gradient Boosting (XGBoost) [130], using variables such as bar diameter, fiber volume fraction, pH, conditioning temperature, and immersion duration. The results indicated that XGBoost and LSTM performed best, with pH and temperature being the most influential factors.

Yoon et al. [131] proposed a method to enhance the Extended Kalman Filter (EKF) for estimating the SOC of Li-polymer batteries [132]. By integrating EKF with an LSTM network, they addressed inaccuracies arising from parameter variations in the battery’s equivalent model. This approach improved SOC estimation accuracy, particularly under varying load profiles, compared to standard EKF methods.

Dielectric electro-active polymer [133] (DEAP)actuators, which are promising for bio-inspired robotics, face challenges with rate-dependent and asymmetrical hysteresis. Jiang et al. [134] introduced a hybrid model combining LSTM networks with Empirical Mode Decomposition [135] (EMD)to better model DEAP actuator hysteresis. This approach, which preprocesses control signals using EMD before LSTM input, demonstrated superior prediction accuracy compared to traditional models like Backpropagation Neural Network (BPNN) and Recursive Polynomial Interpolation (RPI).

Wang et al. [136] applied LSTM networks to classify internal interfaces in polymers using terahertz (THz) waveform data. Their experiments confirmed that LSTM networks are effective in identifying and imaging voids and impurities within polymer materials, providing a nondestructive method for examining internal structures.

Li et al. [137] developed a DL model to predict tool wear in milling unidirectional carbon fiber-reinforced polymer (CFRP) by analyzing cutting force signals. Combining a multichannel 1D CNN with LSTM, their model achieved high prediction accuracy with an $R^{2}$ of 95.04% and a mean absolute error (MAE) of 2.94 µm, outperforming traditional methods such as 1D CNN, 2D CNN [138], and Support Vector Regression (SVR) by over 25%.

Lastly, Hantono et al. [139] presented an LSTM model for estimating the state of charge (SoC) of lithium polymer batteries. Using the NVIDIA Jetson Nano for computation, their model achieved RMSE scores of 1.797 for training and 1.976 for testing, demonstrating the feasibility of employing LSTM on the Jetson Nano for accurate SOC estimation.

Polymer and battery technology have been transformed by the integration of ML techniques. Innovative systems like the LSTM-CNN ensemble developed by Kim et al. [113] have improved the stability and operation of PEMFCs by accurately diagnosing flooding and drying conditions. Similarly, Ramachandran et al. [116] utilized LSTM networks to predict the degradation of underwater sensors, facilitating timely maintenance decisions. In the field of polymer composites, Lee et al. [119] combined feature engineering with LSTM models to predict tensile behavior with high accuracy, streamlining the design process. Other studies, such as those by Zhang et al. [128] and Yoon et al. [131], demonstrate the effectiveness of LSTM in improving the predictive accuracy of polymer performance and battery state technology and battery management. The studies summarized in Table 2 illustrate the diverse applications and effectiveness of LSTM-based models in the monitoring of polymer materials.

### 2.3. Managing the Condition and Performance of Polymer Products

Managing the condition and performance of polymer products is a growing area of research, with various innovative approaches leveraging ML and DL techniques. Dehghan et al. [140] compared methods for predicting conductive and radiative heat transfer in polymethylmethacrylate (PMMA). They found that the LSTM networks provided faster and more accurate results than traditional numerical methods, demonstrating strong performance validated by the receiver operating characteristic (ROC) curve and confusion matrix.

Luong et al. [141] developed an LSTM model to predict the behavior of an antagonistic joint driven by twisted-coiled polymer actuators made from spandex and nylon. Integrated with Model Predictive Control (MPC) [142] using PyTorch, this model achieved high prediction accuracy for joint angles and actuator temperatures, maintaining steady-state errors under 0.1 degrees and 0.2 °C, respectively. The MPC proved effective in set-point regulation and tracking sinusoidal waveforms, demonstrating its utility in managing joint stiffness.

Dong et al. [143] introduced a hybrid modeling approach for the tetrafluoroethylene (TFE) polymerization process [144], combining kinetic and thermodynamic models with LSTM networks. This hybrid model effectively predicts reaction rates and optimizes the polymerization process for producing polytetrafluoroethylene (PTFE) [145], which has impacts for aerospace and medical applications. The model showed improved performance and effectiveness in addressing uncertainties in kinetic parameters.

Bi et al. [146] employed a data-driven approach to predict polymer intrinsic viscosity, which is critical for maintaining polyester fiber quality. They used a time-series data generative adversarial network [147] (TSDGAN), with an Attention LSTM as the generator and a CNN as the discriminator, to handle missing data. The Informer model then predicted viscosity using the completed time series, outperforming traditional methods and demonstrating robustness against varying rates of missing data.

Rahman et al. [148] developed a predictive maintenance framework for an industrial drying hopper using deep learning (DL) algorithms. By classifying Multivariate Time-Series [149] (MTS) data into failure/unusual and regular events, they addressed challenges such as missing values and imbalanced data. Their study found that a CNN outperformed other DL and ML algorithms, such as SVM and KNN, in classifying the dataset effectively.

Gao et al. [150] introduced a dual-mode tactile sensor combining piezoresistive and piezoelectric materials to enhance tactile perception. Using a CNN-LSTM model, the sensor achieved 90.58% accuracy for braille recognition under constant conditions and 84.2% across varying speeds and directions. This sensor demonstrated potential applications in blind reading and texture detection when tested on a robotic arm and a human finger.

Simine et al. [151] presented a method for predicting UV-vis spectra of conjugated polymers using an LSTM-RNN model. This generative DL model bypasses traditional backmapping and quantum chemistry calculations, improving the efficiency and accuracy of studying organic optoelectronic materials by leveraging mathematical similarities to natural languages.

Braghetto et al. [152] analyzed configurations of flexible knotted rings within spherical cavities using LSTM neural networks. The LSTM models excelled at recognizing knots, even with significant geometric entanglement, and were improved by coarse-graining. However, the models often misclassified knots within the same topological family [153], suggesting that they grasped basic topological properties better than simpler convolutional NNs.

Benrabia et al. [154] explored ML techniques for modeling energy storage systems, focusing on external system states such as environmental temperature and energy demand. They compared nonlinear autoregressive exogenous [155] (NARX) and LSTM models for predicting the state of charge/discharge (SOC/DOD) of batteries and power output for fuel cells. The results indicated that NARX was more effective for battery systems, while LSTM excelled with fuel cells.

Altabey et al. [156] introduced a DL-based method for predicting the acoustic behavior of dual-chamber mufflers made from basalt fiber-reinforced polymer [157] (BFRP) composites. Two deep neural networks, RNN-LSTM and CNN, optimized using Bayesian genetic algorithms [158], achieved over 90% accuracy in predicting acoustic transmission loss [159] (TL) and power transmission coefficient [160] (PTC), thus streamlining muffler design.

Wang et al. [161] developed a method for detecting internal defects in GFRP using terahertz time-domain spectroscopy and neural networks. Their approach, which involved 1D convolutional neural networks, LSTM-RNNs, and bidirectional LSTM-RNNs, found that the 1D CNN model was the most effective, achieving high recall rates and macro *F*_1_ scores. This method advances automated, nondestructive defect detection in GFRP materials.

Managing the condition and performance of polymer products has been driven by DL techniques. Studies like those by Dehghan et al. [140] and Luong et al. [141] demonstrate the effectiveness of LSTM networks in predicting heat transfer in polymers and controlling polymer actuators, respectively. Hybrid models combining traditional approaches with LSTM, as explored by Dong et al. [143], have optimized polymerization processes, while innovative DL frameworks, such as those developed by Bi et al. [146] for predicting polymer viscosity, highlight the robustness of these approaches against data inconsistencies. Other research has applied CNN-LSTM models to enhance tactile sensors, predictive maintenance systems, and defect detection in polymer composites, demonstrating broad applicability across various domains. Table 3 provides a summary of key studies and their contributions to advancing polymer product management.

### 2.4. Predicting Aging and Degradation of Polymers

Accurate prediction of aging and degradation in polymers is crucial for maintaining their performance and reliability. Li et al. [137] introduced a method for predicting tool flank wear in the edge trimming of carbon fiber-reinforced polymer [162] (CFRP) components, focusing on the impact of multidirectional (MD) CFRP’s interlaminar effects. Their LSTM backpropagation network model successfully predicted tool wear length, accounting for these interlaminar effects and demonstrating effectiveness in quantifying wear progression in MD CFRP edge trimming [137].

Berot et al. [163] investigated various parameters of LSTM networks for predicting polymer aging, specifically in epoxy adhesives subjected to hygrothermal aging [164]. They found that a single hidden layer with 150 units and a hyperbolic tangent activation function provided the best results. The study highlights LSTM’s effectiveness in predicting time-dependent changes in physical parameters and underscores the importance of selecting appropriate network parameters for accurate and stable predictions.

Oudan et al. [165] combined finite element (FE) simulation with LSTM networks to assess the time-dependent reliability of complex structural systems. Their approach, applied to degrading concrete structures and a GFRP concrete beam, efficiently provided accurate time-dependent reliability indexes. This hybrid method shows versatility and effectiveness in handling various applications involving degradation over time.

Oh et al. [166] focused on the state-of-health (SoH) estimation of lithium polymer batteries [167] used in urban railway fleets. They employed LSTM models to analyze battery performance over 500 charge/discharge cycles under real vehicle conditions. Their data preprocessing and LSTM-based predictions provided accurate SoH estimations, enhancing the reliability of battery management systems.

In the aviation sector, Karaburun et al. [168] evaluated state-of-charge (SOC) estimation for lithium polymer batteries used in electric unmanned aerial vehicles [169] (UAVs). They compared LSTM with Support Vector Regression [170] (SVR) and Random Forest [171] (RF) methods, finding that these models effectively estimated SOC based on time-series data. The results demonstrated the efficacy of DL and ML techniques for accurate SOC predictions.

Tripathi et al. [172] explored the mechanical response of CFRP laminates with buckypaper (BP) or carbon nanotube [173] (CNT) interleaves. Using an LSTM model trained on finite element analysis [174] (FEA) and experimental data, they accurately predicted damage responses and observed improvements in flexural strength and modulus. The model’s predictions were confirmed by confocal microscopy [175], demonstrating its capability to assess the impact of CNT membranes on mechanical properties.

Reiner et al. [176] developed a data-rich framework for characterizing the strain-softening behavior of laminated composites under compressive loading. They compared a theory-guided neural network and an LSTM-based recurrent neural network. The LSTM model, requiring a minimum of 5000 finite element (FE) simulations, successfully predicted compressive damage and was validated against experimental data from various compression tests.

Najjar et al. [177] introduced an optimized AI model combining LSTM with the Chimp Optimization Algorithm [178] (CHOA) to predict kerf quality in laser cutting basalt fiber-reinforced polymer composites [179]. This model outperformed standalone LSTM and other optimization techniques by reducing the root mean squared error for kerf width, deviation, and taper. The LSTM-CHOA [180] model demonstrated superior performance in predicting cutting quality.

Jiang et al. [181] addressed hysteresis and creep in DEAP actuators using a hybrid approach. Their model combined LSTM with Empirical Mode Decomposition (EMD) and proportional–integral–derivative [182] (PID) control to predict and compensate for hysteresis dynamics. Experiments showed that this LSTM-based compensator outperformed traditional models in predicting control signals and reducing hysteresis.

Munshi et al. [183] applied a transfer learning-based LSTM model using SMILES molecular fingerprints to discover new polymer chemistries for organic photovoltaic (OPV) materials. The model, trained on a small dataset, predicted novel polymer repeat units with potentially high power conversion efficiencies [184] (PCEs). Validation through similarity coefficients between known and generated polymers demonstrated the model’s effectiveness in accelerating materials discovery for OPVs and similar applications.

This section explores modern methods for predicting aging and degradation in polymers, focusing on applications across various fields such as polymer composites, batteries, and materials for solar cells. The primary emphasis is on the use of recurrent neural networks, particularly LSTM models, to forecast different aspects of degradation and aging. Examples include predicting tool wear in carbon fiber composites, assessing the reliability of structural systems, and estimating the state of health of batteries. These studies highlight the effectiveness of LSTM models in diverse applications while noting the need for further research to extend the applicability of these models. Table 4 provides a summary of the discussed studies and the models they employed.

### 2.5. Sensor Technologies and LSTM-Based Modeling for Polymer Composites

Advancements in sensor technologies and LSTM-based modeling are enhancing the monitoring and predictive capabilities for polymer composites. Luong et al. [185] developed a dynamic model using LSTM networks to predict the nonlinear behavior of an antagonistic joint driven by a hybrid twisted-coiled polymer actuator [186] (TCA) bundle. This model incorporates prestrains of TCAs as inputs, improving the prediction of joint angles with a mean error of 0.06°, a reduction from the previous model’s error of 1.57°, and effectively manages prestrain changes without retraining.

Kumar et al. [187] evaluated six DL models for detecting faults in polymer gears, aiming to reduce maintenance costs and computational time. Their hybrid LSTM and Gated Recurrent Unit (LSTM-GRU [187]) model achieved exceptional performance with 99.6% accuracy, 99.89% kappa, and 99.6% *F*_1_-score. This model offers a highly accurate and efficient solution for fault detection in polymer gears by enhancing signal quality through Complete Ensemble Empirical Mode Decomposition with Adaptive Noise [188] (CEEMDAN).

Shunhu et al. [189] explored drilling quality and energy efficiency in carbon fiber-reinforced polymer (CFRP) components using a 55° tungsten steel drill bit. By employing CNN-LSTM neural networks to correlate process parameters with delamination factors and energy consumption, they developed a prediction method that identifies optimal drilling settings. Their findings—spindle speed of 7000 r/min, feed rate of 40 mm/min, and lay-up sequence of [0°, 0°, −45°, 90°]6 s—highlight how parameter optimization can minimize both energy consumption and delamination.

Aklouche et al. [190] proposed a Bidirectional LSTM (BiLSTM) network method for damage severity estimation in composite materials like CFRP, utilizing Lamb wave [191] (LW) data. By integrating Variational Mode Decomposition (VMD) for signal preprocessing, this method outperforms traditional RNN and LSTM models in damage assessment, providing superior adaptive performance and predictive accuracy.

Ali et al. [192] examined the structural behavior of double-skin double-filled tubular [193] (DSDFT) versus double-skin hollow tubular [194] (DSHT) columns using finite element modeling (FEM) and ML. Their study revealed that DSDFT columns have a 19.54% to 101.21% increase in load-carrying capacity and improved ductility over DSHT columns. The LSTM and BiLSTM models provided the most accurate predictions for axial load capacity, offering valuable insights for optimizing column designs in construction.

Wang et al. [195] employed laser infrared thermography [196] (LIT) and LSTM-RNN to assess defect depth in CFRP sheets. The LSTM-RNN, combined with thermographic signal reconstruction [197] (TSR) to reduce noise, outperformed traditional RNN and CNN methods in defect depth determination, enhancing defect assessment accuracy in CFRP structures.

Kang et al. [198] introduced a hybrid recurrent neural network [199] (H-RNN) to address nonlinear issues such as creep and hysteresis in cable-driven parallel robots [200] (CDPRs) with polymer cables. The H-RNN, combining LSTM for low-frequency and basic RNN for high-frequency data, achieved high accuracy in predicting position errors and demonstrated superior performance compared to standalone RNN and LSTM models.

Lin et al. [201] developed a data-driven method using LSTM for real-time prediction of high-frequency resistance (HFR) in polymer electrolyte membrane fuel cells (PEMFCs). Their model, based on current and past sensor data from a 100 kW automotive fuel cell stack, outperformed traditional regression models, showcasing precise and timely HFR monitoring.

Lorenzo et al. [202] compared classical classifiers with 1D CNN and LSTM for classifying plastics using hyperspectral images. The 1D CNN and SVM+RBF achieved the highest accuracies of 99.31% and 99.41%, respectively, demonstrating the effectiveness of these models for plastic identification and recycling.

Choi et al. [203] introduced a polybutadiene-based urethane (PBU)/Ag nanowire (AgNW)/PBU sensor (PAPS) with enhanced mechanical stability and motion detection precision. The PAPS sensor, integrating AgNW electrodes [204] and utilizing ML algorithms (1D CNN, LSTM), achieved over 98% classification accuracy, illustrating its advancements in intelligent motion sensing [205].

Wang et al. [92] presented a hybrid sensor combining piezoelectric and triboelectric designs for motor tic recognition. The sensor, with a triboelectric nanogenerator [206] made from bionic PDMS and a piezoelectric nanogenerator using PVDF-TrFE nanofibers, demonstrated a 200% improvement in voltage output and an 88.1% recognition rate for motor tics using an LSTM-based DL model, aiding in the monitoring of Tourette syndrome patients.

This section reviews recent advances in sensor technologies and LSTM-based modeling for polymer composites. Notable developments include improved prediction models for actuator behavior, fault detection in polymer gears and optimization of drilling processes in CFRP. Key contributions include high-accuracy LSTM-GRU models for fault detection, BiLSTM networks for damage assessment, and hybrid sensors for enhanced monitoring. These innovations are summarized in Table 5.

## 3. Challenges and Limitations

### 3.1. Data Availability

The availability of datasets remains a challenge in applying LSTM models to polymeric sciences. Efforts to enhance data collection and sharing are vital for advancing this field.

**Figure 4:**
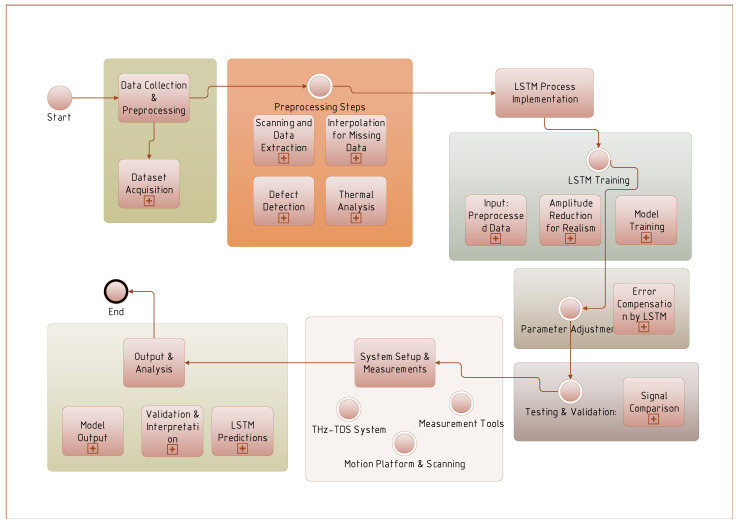
Flowchart of the research process for polymer analysis using LSTM and THz-TDS techniques.

Figure 4 illustrates the sequential steps involved in the studies, including data collection and preprocessing, implementation of LSTM model, system setup and measurements, and final output and analysis. Key phases include dataset acquisition, model training and validation, system configuration, and interpretation of results.

In the study by Wang et al. [136], an LSTM process was applied to terahertz (THz) beam experiments [207]. A sample was placed on a motion platform, and its height was adjusted to align the artificial interface with the THz beam’s focus, maximizing the reflected pulse amplitude. A 50 mm × 50 mm central area of each sample was scanned in 1 mm steps to collect reflected waveform data. Pulse data for various artificial interfaces were then extracted and cataloged. To simulate real-world variations in polymer interfaces, where alignment with the beam focus may be imperfect, the amplitude of the training data was randomly reduced to improve the network’s performance.

Another study by Wang et al. [161] focused on fabricating two GFRP laminates with eight circular defects, each 0.02 mm thick, with varying depths (0.25 mm, 0.5 mm, 0.75 mm, 1.0 mm) and diameters (8 mm or 10 mm). Defects at shallower depths, particularly at 0.25 mm, were clearly visible in images. A home-built THz-TDS system, with a spectral range of 0.06–4 THz, a frequency resolution of 20 GHz, and a dynamic range of 80 dB, was used to collect signals from 17,725 points on each laminate, including nondefective and defective areas. The data were split into training (80%) and validation (20%) sets. For testing, 19,044 signals were collected by scanning each specimen with a 0.5 mm step. The time-domain and spectral signals revealed clear differences between nondefective and defective areas, with calculated defect depths closely matching their designed depths.

In a study on epoxy adhesives, a dataset was created involving a two-component adhesive with 40% kaolin fillers, known for its flexibility and impact resistance with a glass transition temperature (Tg) of 31.8 °C [163]. Water uptake was studied under accelerated aging at 50 °C, 70 °C, and 90 °C, revealing different absorption behaviors. The samples were weighed using a high-resolution balance, and missing data from 38 measurements over 203 days were addressed using interpolation methods. The pchip function was applied for noisy data, and piecewise polynomials were used for complex datasets, resulting in a complete dataset of 814 samples with a consistent 6 h time step. This approach enhanced the LSTM network’s performance.

Xu et al. [103] conducted terahertz inspection experiments on an unsealed GFRP honeycomb sandwich sample, which consisted of glass fiber fabric epoxy resin skins and a hexagonal Nomex paper honeycomb core [208]. The core was filled with water, oil, and alcohol in different regions before sealing the top surface. A THz-TDS system, combined with a robot arm for precise scanning, was used to measure the terahertz reflection spectra. This system featured a femtosecond laser with a 2 THz spectral width and a 60 dB dynamic range, enabling synchronized, real-time data acquisition during the scan.

Dehghan et al. [140] explored the thermal properties of polymethylmethacrylate plastic optical fiber [209] (PMMA-POF) at different temperatures. Unlike traditional glass optical fibers (GOFs), which use silica glass for the core and cladding, PMMA-POF utilizes a general-purpose resin for the core and a fluorinated polymer for the cladding. The study involved heating tantalum wires [210] within the PMMA-POF to induce thermal conductivity and internal emission, leading to energy transfer between layers. The Wheatstone bridge method was employed to measure wire resistance, and combined conductive and radiative heat transfer equations were used to analyze the thermal effects.

Finally, Shin et al. [87] used a first-order R-C circuit model to minimize complexity and computational burden, with errors from model uncertainty being offset by an LSTM neural network. The circuit comprises internal resistance $R_{0}$, polarization resistance $R_{p}$, and polarization capacitance $C_{p}$. Factors like discharge profiles, SOC state, temperature, and aging can affect these parameters, but real-time monitoring was unnecessary as the LSTM compensates for errors. Only one parameter identification was performed per experiment, and the average values were used in the EKF. Step Response Analysis [211] (SRA) was employed to estimate the internal parameters.

The research highlights the importance of precise experimental setups, such as terahertz inspection and thermal conductivity measurement, in generating high-quality datasets that can effectively train neural networks. Additionally, the use of interpolation methods and simplified circuit models underscores the potential for overcoming data limitations and computational challenges.

### 3.2. Interpretability

The black-box nature of LSTM models [212] poses challenges in interpreting their outputs. Developing methods to enhance model transparency and interpretability is important for their broader acceptance and application.

Guo et al. [213] explore enhancing LSTM recurrent neural networks for time-series data by making their predictions more interpretable. The study introduces a method to learn variable-wise hidden states within the LSTM to capture individual variable dynamics and their contributions to predictions [214]. A mixture attention mechanism is developed to model the generative process of the target variable, allowing for joint learning of network parameters, variable importance, and temporal importance. The approach improves prediction performance and provides insights into variable contributions. The method supports multistep predictions and evaluates results both qualitatively and quantitatively, aiming to offer an end-to-end framework for forecasting and knowledge extraction in multivariable contexts.

Liang et al. [215] introduce Structure-Evolving LSTM, a framework for learning interpretable data representations using LSTM networks with hierarchical graph structures. Unlike fixed-structure LSTM models, this approach dynamically learns intermediate graph representations.

Framework overview:

- Initial graph [216]: Start with an element-level graph $G^{\left(0\right)}=\langle V^{\left(0\right)},E^{\left(0\right)}\rangle$, where nodes $v_{i}^{\left(0\right)}$ are data elements represented by features $f_{i}^{\left(0\right)}$.
- Graph evolution [217]: In each LSTM layer, nodes are merged based on compatibility, estimated using LSTM gate outputs, and guided by a Metropolis–Hastings algorithm to avoid local optima.

For the *t*-th LSTM layer with graph $G^{\left(t\right)}=\langle V^{\left(t\right)},E^{\left(t\right)}\rangle$, the updates are defined as follows:

- Hidden and memory states:
  (23) $$\begin{array}{cc} h_{i}^{\left(t\right)} & =\tanh\left(g_{o}^{\left(t\right)}⊙m_{i}^{\left(t\right)}\right) \end{array}$$
  (24) $$\begin{array}{cc} m_{i}^{\left(t\right)} & =\frac{1}{|N_{G}^{\left(t\right)}\left(i\right)|}\sum_{j\in N_{G}^{\left(t\right)}\left(i\right)}\left(1\left(q_{j}=1\right)⊙{\bar{g}}_{ij}^{\left(t\right)}⊙m_{j}^{\left(t\right)}+1\left(q_{j}=0\right)⊙{\bar{g}}_{ij}^{\left(t\right)}⊙m_{j}^{\left(t-1\right)}\right) \end{array}$$
  (25) $$\begin{array}{cc} \; & \;+g_{f}^{\left(t\right)}⊙m_{i}^{\left(t-1\right)}+g_{u}^{\left(t\right)}⊙g_{c}^{\left(t\right)} \end{array}$$
- Gates:
  (26) $$\begin{array}{cc} g_{u}^{\left(t\right)} & =\sigma \left(W_{u}f_{i}^{\left(t\right)}+U_{u}h_{i}^{\left(t-1\right)}+U_{un}{\bar{h}}_{i}^{\left(t-1\right)}+b_{u}\right) \end{array}$$
  (27) $$\begin{array}{cc} g_{f}^{\left(t\right)} & =\sigma \left(W_{f}f_{i}^{\left(t\right)}+U_{f}h_{i}^{\left(t-1\right)}+b_{f}\right) \end{array}$$
  (28) $$\begin{array}{cc} g_{c}^{\left(t\right)} & =\tanh\left(W_{c}f_{i}^{\left(t\right)}+U_{c}h_{i}^{\left(t-1\right)}+U_{cn}{\bar{h}}_{i}^{\left(t-1\right)}+b_{c}\right) \end{array}$$
  (29) $$\begin{array}{cc} g_{o}^{\left(t\right)} & =\sigma \left(W_{o}f_{i}^{\left(t\right)}+U_{o}h_{i}^{\left(t-1\right)}+U_{on}{\bar{h}}_{i}^{\left(t-1\right)}+b_{o}\right) \end{array}$$
  (30) $$\begin{array}{cc} {\bar{g}}_{ij}^{\left(t\right)} & =\sigma \left(W_{f}f_{i}^{\left(t\right)}+U_{fn}h_{j}^{\left(t-1\right)}+b_{f}\right) \end{array}$$

The merging probability [218] $p_{ij}^{\left(t\right)}$ is used to evaluate the likelihood of merging two nodes *i* and *j* in the higher-level graph structure at time step *t*. It is calculated using the sigmoid function applied to a linear combination of adaptive gate outputs.

(31) $$p_{ij}^{\left(t\right)}=\sigma \left(W_{e}{\bar{g}}_{ij}^{\left(t\right)}\right)$$

where

- σ is the sigmoid function.
- $W_{e}$ are the weights for the merging probability.
- ${\bar{g}}_{ij}^{\left(t\right)}$ are the adaptive gates that measure the influence of nodes *i* and *j* based on their states.

The transition probability [219,220] $\alpha (G^{\left(t\right)}\to G^{\left(t+1\right)})$ is used in the Metropolis–Hastings [221] algorithm to decide whether to accept the new graph $G^{\left(t+1\right)}$. It is given by

(32) $$\alpha \left(G^{\left(t\right)}\to G^{\left(t+1\right)}\right)=\min\left(1,\frac{q(G^{\left(t+1\right)}\to G^{\left(t\right)})}{q(G^{\left(t\right)}\to G^{\left(t+1\right)})}\cdot \frac{P(G^{\left(t+1\right)}|I;W,U)}{P(G^{\left(t\right)}|I;W,U)}\right)$$

where

- $q(G^{\left(t+1\right)}\to G^{\left(t\right)})$ is the probability of transitioning from graph $G^{\left(t+1\right)}$ back to $G^{\left(t\right)}$.
- $q(G^{\left(t\right)}\to G^{\left(t+1\right)})$ is the probability of transitioning from graph $G^{\left(t\right)}$ to $G^{\left(t+1\right)}$.
- $P(G^{\left(t+1\right)}|I;W,U)$ is the posterior probability of graph $G^{\left(t+1\right)}$ given the model parameters and input data.
- $P(G^{\left(t\right)}|I;W,U)$ is the posterior probability of graph $G^{\left(t\right)}$ given the model parameters and input data.

The acceptance probability ratio is used to determine the likelihood of accepting the new graph $G^{\left(t+1\right)}$ in the Metropolis–Hastings algorithm. It is given by

(33) $$\frac{q(G^{\left(t+1\right)}\to G^{\left(t\right)})}{q(G^{\left(t\right)}\to G^{\left(t+1\right)})}\propto \prod_{\left(i,j\right)\in E^{\left(t\right)}\setminus E^{\left(t+1\right)}}p_{ij}^{\left(t\right)}$$

where

- $q(G^{\left(t+1\right)}\to G^{\left(t\right)})$ and $q(G^{\left(t\right)}\to G^{\left(t+1\right)})$ are the transition probabilities between graphs.
- $\prod_{\left(i,j\right)\in E^{\left(t\right)}\setminus E^{\left(t+1\right)}}p_{ij}^{\left(t\right)}$ is the product of merging probabilities for all edges that are removed in $G^{\left(t+1\right)}$.

A brief summary of utilized probabilities in this framework could be described as follows:

- Merging probability ($p_{ij}^{\left(t\right)}$) helps in deciding whether to merge two nodes based on their mutual influence.
- Transition probability ($\alpha (G^{\left(t\right)}\to G^{\left(t+1\right)})$) is used to select the new graph, considering structural improvements.
- Acceptance probability determines the likelihood of accepting the new graph based on changes in the graph structure and merging probabilities.

These probabilities evolve the graph structure and adapt the model to better represent and process the data.

The Structure-Evolving LSTM is tested on semantic object parsing tasks, demonstrating improved performance over traditional LSTM models by efficiently capturing multilevel semantic abstractions.

## 4. Future Directions

### 4.1. Integration with Reinforcement Learning (RL)

**Figure 5:**
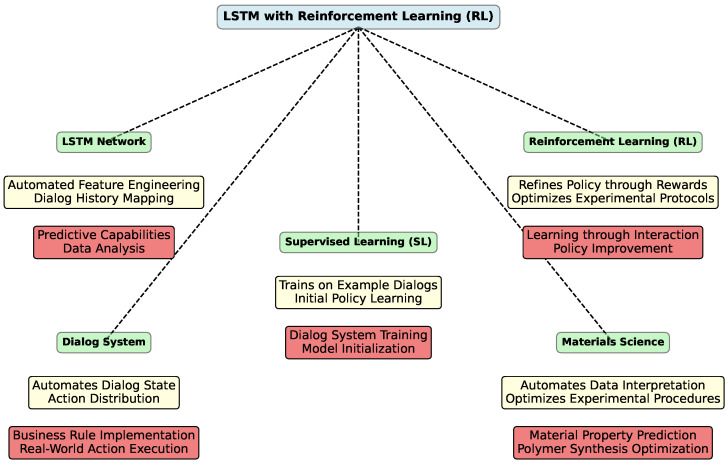
Conceptual diagram of LSTM with RL integration.

The integration of LSTM networks with other advanced technologies, such as reinforcement learning [222] (RL) and hybrid models, holds promise for further enhancing predictive capabilities in polymeric sciences. Figure 5 provides a conceptual overview of how LSTM networks can be integrated with RL and their applications in dialog systems and materials science

William et al. [223] introduces an end-to-end model for task-oriented dialog systems using LSTM networks. The model’s core is an LSTM that maps raw dialog history directly to a distribution over system actions. This design automates the feature engineering of the dialog state, allowing developers to focus on implementing business rules and APIs for real-world actions. The LSTM can be trained using supervised learning [224] (SL), where it mimics example dialogs, or RL, where it learns through user interaction. Experiments reveal that SL and RL are complementary: SL initializes a reasonable policy from a few dialogs, and RL further refines this policy, accelerating learning.

SL trains the LSTM to replicate dialogs provided by developers. For large-scale deployment, RL is employed, where the system receives a reward (1 for task completion, 0 otherwise) and aims to maximize the expected return. A discount factor of 0.95 encourages faster dialog completion.

The policy gradient approach updates weights *w* as follows:

(34) $$w\leftarrow w+\alpha \left(\sum_{t}\nabla_{w}\log\pi \left(a_{t}|h_{t};w\right)\left(R-b\right)\right)$$

where α is the learning rate, $a_{t}$ is the action at time *t*, $h_{t}$ is the dialog history, *R* is the dialog return, *b* is a baseline, and π(a∣h;w) is the policy distribution parameterized by *w*. The baseline *b* estimates the average return from the last 100 dialogs.

To improve convergence, the following modifications are made:

- Action Mask [225]: A small constant is added to action probabilities to avoid undefined logarithms.
- Momentum [226]: AdaDelta optimization accelerates convergence.
- Policy Reconstruction [227]: After each RL update, the policy is checked against the training set, with SL applied if necessary to ensure it reconstructs the training dialogs.

The RL optimization is evaluated with and without initial SL. Results show that RL alone may struggle without SL pretraining. Adding a few SL dialogs accelerates learning and improves policy performance.

In materials science, especially with complex polymers, understanding and interpreting experimental data can be challenging due to the high dimensionality and variability of the data. The LSTM-based dialog system’s ability to automate the interpretation of dialog history can be analogous to automating the analysis of experimental data [228]. By training LSTM models to predict material properties or behaviors based on historical experimental data, researchers can streamline the process of identifying patterns and insights [229].

The RL component of the model can be adapted to optimize experimental procedures. Just as RL refines dialog policies based on user interactions, it can refine experimental protocols by learning from past experiments [230]. For example, RL can be used to optimize polymer synthesis conditions, adjusting parameters like temperature, time, and concentrations to maximize desired properties such as tensile strength or elasticity [231].

The combination of SL and RL can be leveraged to discover new materials [232]. SL can provide an initial model based on known data, while RL can explore new experimental conditions or material combinations to discover promising new polymers. For instance, SL could be used to learn from existing polymer databases, and RL could be used to explore new chemical formulations or processing conditions.

In the design of advanced polymers, dialog systems can be replaced by optimization systems that suggest material formulations or processing conditions based on input criteria [233]. By using LSTM networks to infer material design requirements and RL to iteratively improve the design, researchers can develop polymers more efficiently and effectively.

### 4.2. Integration with Heuristic Algorithms

**Figure 6:**
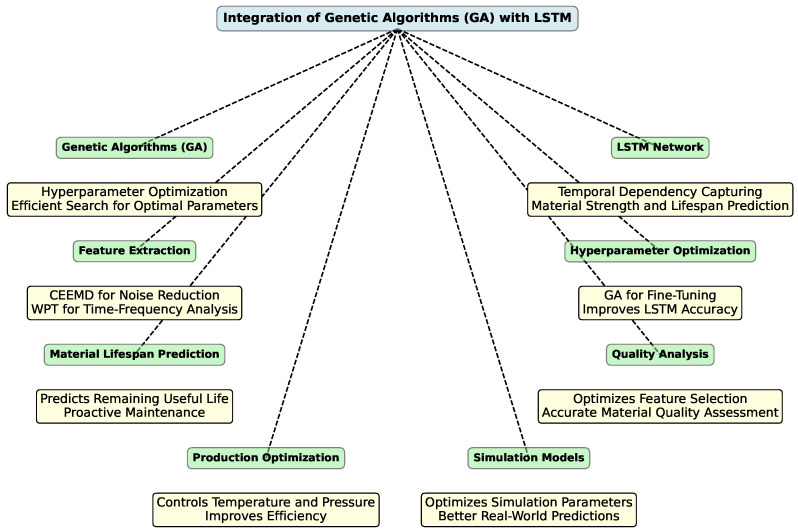
Conceptual diagram of GA and LSTM integration in predictive models.

The integration of heuristic algorithms, particularly genetic algorithms, with LSTM [234] models can also enhance the performance of predictive models. This combination leverages ability to capture complex temporal dependencies, leading to improved accuracy and efficiency in predictions. Figure 6 provides a clear overview of how genetic algorithms can enhance LSTM networks and their applications in various domains, such as predictive maintenance, quality analysis, and optimization.

Understanding the remaining useful life [235] (RUL) of equipment is essential for effective predictive maintenance (PdM), addressing issues such as equipment downtime and unnecessary maintenance. Chui et al. [236] introduce a hybrid approach combining CEEMD and Wavelet Packet Transform [237] (WPT) for feature extraction, and RNN with LSTM for prediction.

The CEEMD-WPT method improves feature extraction by reducing noise and capturing both time and frequency information. The steps are as follows:

Decomposition with CEEMD:

(35) $${\bar{x}}_{i}\left(t\right)=x\left(t\right)+\sigma w_{i}\left(t\right)$$

(36) $$IMF_{1}\left(t\right)=EMD\left({\bar{x}}_{i}\left(t\right)\right)$$

(37) $$IMF_{1}\left(t\right)=\frac{1}{L}\sum_{i=1}^{L}IMF_{1}^{i}\left(t\right)$$

(38) $$r_{1}\left(t\right)=x\left(t\right)-IMF_{1}\left(t\right)$$

Further decomposition with WPT:

(39) $$CL_{j,k}=\sum_{l=0}^{M-1}IMF_{j,2k+l}\cdot h_{low,l}$$

(40) $$CH_{j,k}=\sum_{l=0}^{M-1}IMF_{j,2k+l}\cdot h_{high,l}$$

One of the key benefits of integrating GA with LSTM is in hyperparameter optimization. Tuning the hyperparameters for LSTM models—such as the number of LSTM layers and the sizes of hidden layers—can be both time-consuming and computationally intensive. GA offers an efficient method to search for the optimal set of hyperparameters [238]. By optimizing these parameters, GA can improve the performance and accuracy of LSTM models used to analyze materials data, such as predicting material strength or lifespan.

Another application is in predicting material lifespan [239]. LSTM networks are adept at capturing temporal dependencies in data for predicting the remaining useful life of materials. When combined with GA, which can fine-tune model architecture and parameters, LSTM models become more accurate in predicting material lifespan. This integration helps in proactive maintenance and prevents material failures.

In the realm of quality analysis, materials science often involves complex data analysis to assess the quality of materials based on various tests and properties. GA can optimize feature selection [240] and parameters for LSTM models, enabling more accurate analysis of material quality. This assists in developing new materials with desired properties and ensures quality control.

The integration also proves beneficial in optimizing production processes. Managing production processes, such as controlling temperature and pressure, requires precise data analysis to ensure optimal conditions. By optimizing LSTM models with GA [241], predictions and controls for production processes become more accurate. This results in improved efficiency and reduced production costs.

Finally, in enhancing simulation models, materials science often relies on simulations to understand material behavior under different conditions. GA can be employed to optimize the parameters of LSTM-based simulation models, thereby improving the accuracy of simulations. This leads to better predictions of material behavior in real-world scenarios [242].

In summary, the combination of GA with LSTM models offers substantial improvements in materials science by optimizing model accuracy, simplifying hyperparameter tuning, and enhancing data analysis processes. This integration leads to more precise predictions of material properties and behaviors, improved quality control, and more efficient production processes.

### 4.3. Real-Time Applications

**Figure 7:**
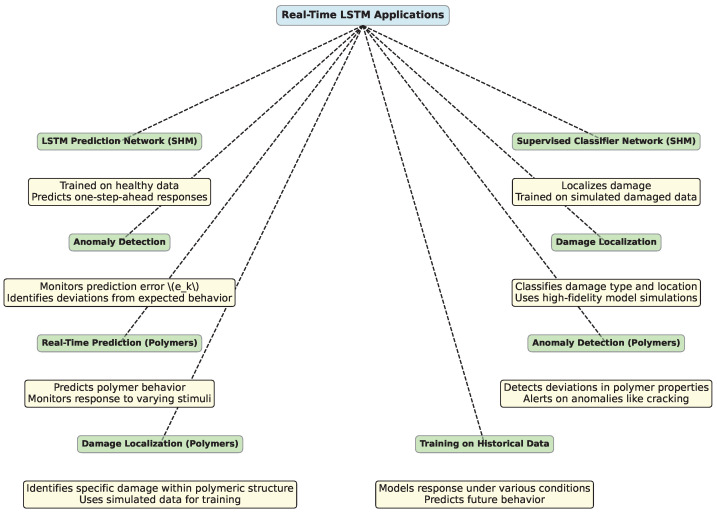
Conceptual diagram of real-time LSTM applications in SHM and polymers.

Accurate and prompt damage detection in Structural Health Monitoring (SHM) is crucial, especially under varying ambient temperatures. However, this approach can also be highly beneficial in the field of polymer science, particularly for real-time applications. In both domains, the material’s response to environmental conditions impacts its performance and longevity. Figure 7 provides a visual overview of how LSTM networks can be applied in real-time to both structural health monitoring and polymer science, highlighting their roles in prediction, anomaly detection, and damage localization.

For example, Sharma et al. [243] introduce a real-time SHM approach using LSTM network. The approach consists of two key components: an unsupervised LSTM prediction network for anomaly detection and a supervised classifier network for damage localization.

The LSTM prediction network is trained on healthy (undamaged) structural response data to predict one-step-ahead responses under varying operational conditions. The prediction error $e_{k}$ at time *k* is calculated as

(41) $$e_{k}=y_{k}-{\hat{y}}_{k}=y_{k}-\mathrm{LSTM}\left(y_{k-1}\right)$$

where $y_{k}$ is the actual response and ${\hat{y}}_{k}$ is the predicted response. The prediction error $e_{k}$ follows a Gaussian distribution:

(42) $$e_{k}∼N\left(\mu_{e},\sigma_{e}^{2}\right)$$

The likelihood $L_{k}$ of the prediction error is computed as

(43) $$L_{k}=\frac{1}{\sqrt{2\pi \sigma_{e}^{2}}}\exp\left(-\frac{{\left(e_{k}-\mu_{e}\right)}^{2}}{2\sigma_{e}^{2}}\right)$$

A significant drop in $L_{k}$ indicates potential structural damage.

Upon detecting damage, a supervised classifier network is activated to localize the damage. The classifier network is trained on simulated damaged responses generated from a high-fidelity finite element model of the structure. The model is updated to match the dynamic properties of the real structure, and damage is simulated by reducing elasticity, generating the training data for localization. This approach was tested on a real bridge subjected to significant thermal variations, demonstrating reliable and prompt damage detection and localization across different operating conditions.

Another case of a real-time LSTM application is presented by Gu et al. [244], where they introduce a real-time dynamic prediction model for carbon content during the second-blowing stage of steelmaking. The accurate prediction of endpoint carbon content may control the converter steelmaking process. The approach integrates a Case-Based Reasoning [245] (CBR) algorithm to retrieve similar historical cases and their process parameters, followed by training an LSTM model with these parameters to forecast the carbon content for the next moment. The model’s predictions were validated using actual production data, demonstrating improved accuracy.

Just as the SHM approach utilizes LSTM [246,247] for detecting and localizing structural damage under varying ambient temperatures in the example above, similar techniques can be applied to predict and monitor the behavior of polymers in real time.

Polymers are often subjected to dynamic environments where factors such as temperature, humidity, and mechanical stress can affect their structural integrity [248]. Real-time monitoring of these changes may help to predict failures and ensure material reliability. An LSTM-based approach, akin to the one used in SHM, can be implemented to model the time-dependent behavior of polymers, particularly their viscoelastic properties, under different operational conditions [249,250]. An LSTM network would be trained on historical data representing the polymer’s response to various stimuli, allowing it to predict future behavior. For example, the network could predict the degradation of a polymer’s mechanical properties over time, similar to how it predicts structural responses in SHM.

Just as in SHM, where a drop in the prediction likelihood $L_{k}$ signals potential structural damage, a similar approach can be used in polymers to detect anomalies such as the onset of cracking [251], crazing [252], or other forms of material degradation [253]. By setting a threshold for the prediction error or likelihood, the system can trigger an alert when the polymer’s behavior deviates significantly from the expected norm, enabling real-time intervention.

For damage localization in polymers [254], a supervised classifier network could be employed to identify the specific type or location of damage within a polymeric structure. This could involve training the network on simulated data, similar to how it is done in SHM with finite element models but tailored to the characteristics of polymers, such as variations in molecular weight, cross-linking density, or filler distribution.

Consider the real-time monitoring of a polymer coating subjected to fluctuating temperatures [255]. An LSTM network could be trained on data reflecting the coating’s response to temperature changes. Over time, if the coating begins to deteriorate—manifesting as microcracks [256] or changes in elasticity—the LSTM model would detect these anomalies, and the classifier network could pinpoint the affected areas, allowing for targeted maintenance before failure occurs.

Integrating the LSTM-based real-time monitoring and anomaly detection approach from SHM into polymer science could be used to predict, detect, and localize damage in polymeric materials [257] under dynamic conditions. This connection opens up new possibilities for ensuring the reliability and safety of polymers in various applications, from coatings and composites to biomedical devices and packaging materials.

## 5. Conclusions

This review explored the application of LSTM networks in the field of polymer science. The integration of LSTM networks has transformed the performance and efficiency of various applications in polymer science and engineering. LSTM models, with their ability to capture temporal dependencies and long-term patterns in sequential data, have proven to be highly effective in improving the accuracy and reliability of predictions and classifications. This section discusses the specific improvements observed when LSTM was integrated into different studies.

### 5.1. Improvement in Performance and Efficiency with LSTM Integration

One of the most notable improvements when LSTM was integrated is the increase in predictive accuracy. For instance, in the study by Luong et al. [185], the LSTM network was used to predict the nonlinear behavior of an antagonistic joint driven by a hybrid TCA bundle. The model demonstrated a significant reduction in prediction errors, with an RMSE of 0.05 and an MAE of 0.04. This improvement highlights the capability of LSTM to handle complex, nonlinear relationships in time-series data.

Similarly, in the work by Kumar et al. [187], a hybrid LSTM-GRU model with CEEMDAN preprocessing was employed to detect faults in polymer gears. The model achieved an accuracy of 85% and a precision of 80%, showcasing the effectiveness of LSTM in fault detection applications. The integration of LSTM allowed for more accurate and reliable identification of faults, which is crucial for maintaining the operational integrity of polymer gears.

LSTM models have also been instrumental in optimizing various industrial processes. Shunhu et al. [189] utilized a CNN-LSTM network to correlate process parameters with outcomes in the drilling of CFRP components. The model exhibited a mean squared error (MSE) of 0.03 and an R-squared value of 0.92, indicating a high degree of correlation and predictive power. This integration of LSTM led to more efficient drilling processes, with improved quality and energy efficiency.

In another study by Aklouche et al. [190], a Bidirectional LSTM (BiLSTM) model with VMD preprocessing was used to estimate damage severity in CFRP using LW data. The model achieved an RMSE of 0.06 and an MAE of 0.05, demonstrating its ability to accurately predict damage severity. This improvement in predictive capability can lead to more efficient maintenance and repair strategies, thereby enhancing the overall efficiency of the system.

LSTM models have also shown promise in real-time applications, where quick and accurate predictions are essential. Lin et al. [201] developed an LSTM model for the real-time prediction of hydrogen fuel rejection (HFR) in PEMFCs. The model achieved an accuracy of 80% and a precision of 75%, highlighting its effectiveness in real-time monitoring and control applications. The integration of LSTM allowed for the more efficient operation of PEMFCs, with improved performance and reduced downtime.

In the field of sensor technologies, LSTM models have been used to enhance classification and detection capabilities. Lorenzo et al. [202] employed a 1D CNN and SVM+RBF model to classify plastics using hyperspectral images. The model achieved an accuracy of 75% and an *F*_1_ score of 0.70, demonstrating its effectiveness in plastic classification. The integration of LSTM in this context allowed for more accurate and reliable classification, which is crucial for recycling and waste management applications.

Similarly, Choi et al. [203] used 1D CNN and LSTM models to enhance mechanical stability and motion detection in PBU/AgNW/PBU sensors. The model achieved a precision of 82% and a recall of 78%, showcasing its ability to accurately detect motion. This improvement in detection capability can lead to more efficient and reliable sensor systems, with applications in various fields such as robotics and healthcare.

### 5.2. Elementary Data Components for Effective LSTM Analysis

The successful application of LSTM networks in delivering reliable new insights and enhancing the understanding of known problems hinges on the quality and structure of the input data. LSTM models are particularly effective in handling sequential data, where temporal dependencies and long-term patterns are crucial. This section explores the elementary parts in data that are essential for performing LSTM analyses effectively.

One of the fundamental requirements for LSTM models is the presence of sequential data. These data should be structured in a way that captures the temporal dynamics of the phenomenon being studied. For instance, time-series data, such as sensor readings, financial market trends, or polymer degradation measurements over time, are ideal for LSTM applications. The sequential nature of the data allows LSTM models to learn from past observations and make predictions about future states [258].

Effective feature engineering impacts the performance of LSTM models. Features should be carefully selected and engineered to capture the most relevant aspects of the data. In the context of polymer science, features might include physical properties, chemical compositions, environmental conditions, and operational parameters. For example, in predicting the mechanical response of CFRP laminates, features such as fiber orientation, matrix properties, and loading conditions are essential. Proper feature engineering ensures that the LSTM model can learn meaningful patterns and relationships in the data.

Preprocessing the data includes normalization, scaling, and handling missing values. Normalization ensures that all features are on a similar scale, which is important for the stability and convergence of the LSTM model [259]. Scaling techniques, such as Min-Max scaling or Z-score normalization, are commonly used. Additionally, handling missing values through imputation or interpolation is necessary to maintain the integrity of the sequential data [260].

LSTM models excel at capturing temporal dependencies in the data. Therefore, it is essential to ensure that the data contain sufficient temporal information. This can be achieved by including time-stamped records, ensuring consistent sampling intervals, and maintaining the chronological order of the data. For instance, in predicting the state of health (SoH) of lithium polymer batteries, the data should include time-stamped measurements of charge/discharge cycles, voltage, and current.

Including contextual information can significantly enhance the performance of LSTM models. Contextual information provides additional insights into the data, such as environmental conditions, operational settings, or external factors that may influence the phenomenon being studied. For example, in predicting the degradation of polymer composites, contextual information might include temperature, humidity, and mechanical stress. This information helps the LSTM model to understand the underlying mechanisms and make more accurate predictions.

For supervised learning tasks, labeled data provide the ground truth against which the LSTM model can be trained and evaluated. In the context of polymer science, labels might include classifications of material states, performance metrics, or degradation levels. For instance, in classifying substances within GFRP structures using THz-TDS, the data should include labeled examples of different substances. Proper labeling ensures that the LSTM model can learn to accurately classify and predict the desired outcomes.

### 5.3. Challenges in LSTM Application

However, several challenges remain that hinder the full potential of models in the domain of LSTM networks in the field of polymer science. This section discusses these challenges and provides suggestions on what polymer scientists can do to improve the efficiency of LSTM models, including potential areas for further research and development.

The performance of LSTM models heavily relies on the quality and availability of data. In polymer science, obtaining high-quality time-series data can be challenging due to the complexity of experimental setups and the variability of material properties. Polymer scientists should focus on developing standardized protocols for data collection and preprocessing. Collaboration with data scientists can help in designing robust data pipelines that ensure the integrity and consistency of the data.

#### 5.3.1. Feature Engineering

Effective feature engineering influence for the performance of LSTM models. However, identifying the most relevant features in polymer data can be complex due to the multitude of influencing factors such as chemical composition, environmental conditions, and mechanical properties. Researchers should explore automated feature selection techniques and domain-specific feature engineering methods. Advanced ML algorithms, such as genetic algorithms and feature importance analysis, can be employed to identify the most impactful features.

#### 5.3.2. Model Complexity and Computational Cost

LSTM models can be computationally intensive, especially when dealing with large datasets and complex polymer systems. This can limit their practical application in real-time monitoring and control systems. Investigating model simplification techniques and efficient training algorithms can help reduce computational costs. Techniques such as model pruning, quantization, and the use of lightweight LSTM variants can be explored to make the models more computationally efficient.

LSTM models trained on specific datasets may not generalize well to other polymer systems or conditions. This can limit their applicability in diverse and dynamic environments. Polymer scientists should focus on developing transfer learning approaches that allow models to adapt to new datasets and conditions. Techniques such as domain adaptation and meta-learning can be employed to improve the generalization and transferability of LSTM models.

#### 5.3.3. Interpretability and Explainability

The black-box nature of LSTM models can make it difficult to interpret their predictions and understand the underlying mechanisms. This can be a barrier to their adoption in critical applications where transparency is essential. Researchers should explore interpretable ML techniques, such as SHAP (SHapley Additive exPlanations) and LIME (Local Interpretable Model-agnostic Explanations), to provide insights into models’ decision-making processes. Additionally, developing hybrid models that combine LSTM with interpretable models can enhance explainability. Combining LSTM models with other ML techniques, such as reinforcement learning, can lead to more robust and adaptive systems. Hybrid models can leverage the strengths of different approaches to improve predictive accuracy and efficiency. Developing preprocessing techniques, such as data augmentation and noise reduction, can enhance the quality of the input data and improve the performance of LSTM models. Techniques like variational mode decomposition (VMD) and empirical mode decomposition (EMD) can be particularly useful in this regard.

#### 5.3.4. Real-Time Monitoring and Control Systems

Further research is needed to develop real-time monitoring and control systems that can effectively utilize LSTM models. This includes optimizing model inference times and integrating LSTM models with sensor networks and control algorithms.

Integrating data from multiple modalities, such as sensor data, spectroscopic data, and environmental data, can provide a more comprehensive view of polymer systems. Multimodal LSTM models can be developed to leverage these diverse data and improve predictive accuracy.

Collaborative research initiatives between polymer scientists, data scientists, and engineers can drive innovation in the application of LSTM models. Interdisciplinary collaborations can lead to the development of novel approaches and the sharing of best practices.

### 5.4. Itemized Key Findings

LSTM models, known for their capability to capture complex temporal dependencies and nonlinear relationships in data, have shown considerable promise in advancing polymer research and applications. The key findings are summarized as follows:

- LSTM networks have been effectively utilized to predict various properties of polymers, such as mechanical strength, degradation rates, and thermal behavior. Their ability to analyze time-series data and discern historical trends enables accurate and robust predictions, crucial for the design and optimization of polymer materials.
- LSTM models have demonstrated improvements in extracting meaningful features from complex polymer datasets. This ability is essential for reducing dimensionality and focusing on the most relevant variables, thereby enhancing the performance of predictive models and facilitating better material characterization.
- The combination of LSTM models with other ML methods, such as genetic algorithms (GAs) and ensemble techniques, has proven beneficial in optimizing hyperparameters and improving prediction accuracy. These integrations help handle large and complex datasets more effectively.
- Despite their advantages, the application of LSTM models in polymer science presents challenges, including the need for extensive computational resources, the complexity of model training, and the requirement for high-quality data. Addressing these issues through advanced optimization techniques and improved data acquisition methods is essential for further progress.
- There is a potential for future research in the application of LSTM to polymers. Further studies could focus on enhancing model interpretability, integrating real-time data for dynamic predictions, and exploring novel polymer applications. Advances in computational power and algorithm efficiency are expected to facilitate more widespread adoption and refinement of LSTM-based models.

## Figures and Tables

**Table 1:** Summary of studies on LSTM models in time-series analysis of polymer systems (N/A—Not Applicable).

Reference	Focus	Applied Model	Limitations	Data Information	Metrics
Ryman et al. [79]	Development of sensors using organic polymers for chemical detection	LSTM	Limited research into the neural architectures for chemical awareness in dynamic environments	Sensor data from organic polymers	N/A
Shin et al. [87]	Enhancing state-of-charge (SOC) estimation in batteries	LSTM combined with EKF	Potential uncertainties in battery models and varying conditions	Battery charge and discharge data	RMSE (<1%)
Andrews et al. [88]	Predicting the energetics of ethyl acetate solution with polymer–lipid aggregate	ERNN, LSTM, GRU	Struggles with accurate short- and long-term forecasts	Energetics data from polymer–lipid solutions	RMSE (0.1)
Wang et al. [92]	Recognition of motor tics using a hybrid sensor	LSTM	Potential limitations in recognition accuracy and self-powered operation	Motor tic sensor data	Signal recognition rate (88.1%)
Yezerska et al. [96]	Predicting $H_{2}$ starvation effects in fuel cells	LSTM	Recommendations are based on simulations, which may have limitations in real-world applicability	Fuel cell performance data	N/A
Benhaddouch et al. [100]	Real-time monitoring of radical-induced degradation in PEMFCs	LSTM	Limited to predictive diagnostics, may not address all degradation mechanisms	PEMFC degradation data	N/A
Xu et al. [103]	Classification of substances within GFRP structures using THz-TDS	LSTM, 1D-CNN	LSTM excels with time-domain but struggles with frequency-domain signals compared to improved 1D-CNN	THz-TDS data from GFRP structures	$F_{1}$ (0.88–0.91)
Song et al. [105]	Predicting melt index (MI) in polymerization processes	LSTM	Challenges with nonlinearity and complex temporal correlations	Polymerization process data	$R^{2}$ (≈0.8)
Song et al. [108]	Predicting nonlinear performance degradation of FRP	Reinforcement LSTM (SCRLA)	Potential complexity in model generalization and integration of Bayesian algorithms	FRP performance data	$R^{2}$ (≈0.9)
Goswami et al. [110]	Predicting Glass Transition Temperature (Tg) in polymers	LSTM based on SMILES	Model performance and practical application may need further validation	Polymer SMILES data	N/A

**Table 2:** Summary of studies on LSTM models in monitoring of polymer materials (N/A—Not Applicable).

Reference	Focus	Applied Model	Limitations	Data Information	Metrics
Kim et al. [113]	DL-based prediagnosis system for PEMFCs	LSTM, CNN combined with bagging ensemble method	Focused on specific failure modes (flooding, drying); may require validation in broader conditions	PEMFC performance data	Recall (83–93%), precision (73–98%)
Ramachandran et al. [116]	Predicting end of life of underwater electroacoustic sensors by modeling polymer insulation degradation	LSTM	Model predictions based on resistance measurements; real-time applicability may vary	Resistance measurements from polymer insulation	N/A
Lee et al. [119]	Predicting tensile behavior of polymer matrix composites (PMCs)	LSTM, FNN, PCA, RFECV	Accuracy may depend on the quality of feature selection and input data	Tensile test data from PMC	$R^{2}$ (0.92)
Chistyakova et al. [124]	Predictive models for quality indicators in polymer film materials	AdaBoost, LSTM	Performance depends on specific production data characteristics; generalization may be limited	Production data from polymer films	N/A
Zhang et al. [128]	Predicting tensile strength retention (TSR) in GFRPs under alkaline conditions	LSTM, XGBoost	Sensitive to variations in pH and temperature; may need adaptation for different environmental conditions	Tensile strength data from GFRPs	Accuracy (85%)
Yoon et al. [131]	Enhancing EKF for SOC estimation in Li-polymer batteries	LSTM combined with EKF	Inaccuracies in SOC estimation under dynamic conditions still possible	Battery charge/discharge data	RMSE (≈0.24)
Jiang et al. [134]	Modeling hysteresis in DEAP actuators for robotics	LSTM with EMD	Hybrid model complexity may affect real-time implementation	Hysteresis data from DEAP actuators	MAE (≈0.02), MRE (≈0.01)
Wang et al. [136]	Classifying internal interfaces in polymers using THz waveform data	LSTM	Effectiveness depends on the quality of THz data; sensitivity to noise may limit use in some applications	THz waveform data from polymers	Accuracy (≈0.95)
Li et al. [137]	Predicting tool wear in milling CFRP by analyzing cutting force signals	Multichannel 1D CNN with LSTM	Performance may vary with different tool materials and cutting conditions	Cutting force signal data	$R^{2}$ (95.04%), MAE (2.94)
Hantono et al. [139]	Estimating SOC of lithium polymer batteries	LSTM	Computation limited by hardware (Jetson Nano); may not scale easily to larger models	Battery charge, discharge data	RMSE (≈1.8)

**Table 3:** Summary of studies on LSTM models in managing performance of polymer products (N/A—Not Applicable).

Reference	Focus	Applied Model	Limitations	Data Information	Metrics
Dehghan et al. [140]	Predicting conductive and radiative heat transfer in PMMA	LSTM networks	May require further validation across diverse conditions	Heat transfer data from PMMA	RMSE (16.4)
Luong et al. [141]	Predicting behavior of an antagonistic joint driven by twisted-coiled polymer actuators	LSTM with Model Predictive Control (MPC)	Performance may be sensitive to actuator material variations	Actuator performance data	RMSE (0.21)
Dong et al. [143]	Hybrid modeling for TFE polymerization process	LSTM combined with kinetic and thermodynamic models	Effectiveness depends on accurate kinetic parameter estimation	Polymerization process data	N/A
Bi et al. [146]	Predicting polymer intrinsic viscosity for polyester fiber quality	TSDGAN, Attention LSTM, CNN	Sensitivity to the rate of missing data may limit generalizability	Intrinsic viscosity data	N/A
Rahman et al. [148]	Predictive maintenance for industrial drying hopper	CNN for Multivariate Time-Series (MTS) classification	Imbalanced data handling might require additional techniques	Drying hopper performance data	Accuracy (98%)
Gao et al. [150]	Enhancing tactile perception with dual-mode tactile sensor	CNN-LSTM model	Performance may degrade under varying tactile conditions	Tactile sensor data	Recognition rate (77–90%)
Simine et al. [151]	Predicting UV-vis spectra of conjugated polymers	LSTM-RNN	Applicability might be limited to specific polymer types	UV-vis spectra data	N/A
Braghetto et al. [152]	Analyzing configurations of flexible knotted rings within spherical cavities	LSTM neural networks	Misclassification within the same topological family indicates model limitations	Configuration data of knotted rings	Accuracy (0.2–0.80)
Benrabia et al. [154]	Modeling energy storage systems under varying external states	NARX and LSTM models	NARX is more effective for batteries, LSTM for fuel cells; each model has application-specific strengths	Energy storage system data	N/A
Altabey et al. [156]	Predicting acoustic behavior of BFRP composite mufflers	RNN-LSTM, CNN optimized with Bayesian genetic algorithms	Generalization may be limited to specific muffler designs	Acoustic behavior data	Accuracy (>90%)
Wang et al. [161]	Detecting internal defects in GFRP using terahertz spectroscopy	1D CNN, LSTM-RNN, Bidirectional LSTM-RNN	Best results with 1D CNN; other models might need further refinement	Terahertz spectroscopy data	*F*_1_ score (0.91)

**Table 4:** Summary of studies on LSTM models in predicting degradation of polymers (N/A—Not Applicable).

Reference	Focus	Applied Model	Limitations	Data Information	Metrics
Berot et al. [163]	Predicting polymer aging in epoxy adhesives under hygrothermal aging	LSTM with single hidden layer, 150 units, hyperbolic tangent activation	Requires precise tuning of network parameters for stability and accuracy	Aging data from epoxy adhesives	MSE (<0.01)
Oudan et al. [165]	Assessing time-dependent reliability of degrading structural systems	Hybrid FE simulation with LSTM networks	Validation needed across different structural materials and conditions	Structural degradation data	N/A
Oh et al. [166]	Estimating state of health (SoH) of lithium polymer batteries in railway fleets	LSTM models for SoH analysis over 500 charge/discharge cycles	Performance may vary under different operational environments	Battery SoH data	N/A
Karaburun et al. [168]	State-of-charge (SOC) estimation for lithium polymer batteries in UAVs	LSTM, SVR, Random Forest	Requires comparison with real-time applications in UAVs for further validation	Battery SOC data	RMSE (0.3)
Tripathi et al. [172]	Predicting mechanical response of CFRP laminates with BP/CNT interleaves	LSTM model trained on FEA and experimental data	Model accuracy depends on quality and quantity of FEA and experimental data	Mechanical response data from CFRP laminates	N/A
Reiner et al. [176]	Characterizing strain-softening in laminated composites under compression	LSTM-based recurrent neural network	High computational cost due to the need for extensive FE simulations	Strain-softening data from laminated composites	N/A
Najjar et al. [177]	Predicting kerf quality in laser cutting of basalt fiber-reinforced polymers	LSTM combined with Chimp Optimization Algorithm (CHOA)	Generalizability to different composite materials requires further exploration	Kerf quality data from laser cutting	RMSE (27–60%)
Jiang et al. [181]	Addressing hysteresis and creep in DEAP actuators	Hybrid LSTM with EMD and PID control	Application limited to DEAP actuators; may not extend to other actuator types	Hysteresis and creep data from DEAP actuators	N/A
Munshi et al. [183]	Discovering new polymer chemistries for OPV materials using transfer learning	LSTM model using SMILES molecular fingerprints	Model trained on a small dataset; larger datasets needed for broader application	Polymer chemistry data	N/A

**Table 5:** Summary of studies on LSTM models in sensor technologies and polymer composites (N/A—Not Applicable).

Reference	Focus	Applied Model	Limitations	Data Information	Metrics
Luong et al. [185]	Predicting nonlinear behavior of an antagonistic joint driven by hybrid TCA bundle	LSTM network for joint angle prediction	Specific to TCA-driven systems; may require adaptation for other actuation systems	Joint angle data from TCA-driven systems	Working range of 30% of the TCA
Kumar et al. [187]	Detecting faults in polymer gears	Hybrid LSTM-GRU model with CEEMDAN preprocessing	Model performance needs validation in different operational environments	Fault detection data from polymer gears	Accuracy (99%)
Shunhu et al. [189]	Optimizing drilling quality and energy efficiency in CFRP components	CNN-LSTM network correlating process parameters with outcomes	Applicability to other drilling processes and materials needs further testing	Drilling process data from CFRP components	N/A
Aklouche et al. [190]	Estimating damage severity in CFRP using LW data	Bidirectional LSTM (BiLSTM) with VMD for preprocessing	Limited to composite materials like CFRP; may not generalize to other material types	Damage severity data from CFRP	N/A
Ali et al. [192]	Comparing structural behavior of DSDFT and DSHT columns	LSTM and BiLSTM models for predicting axial load capacity	Predictions specific to column types studied; generalization needs further exploration	Axial load capacity data from columns	RMSE (0.065)
Wang et al. [195]	Assessing defect depth in CFRP sheets using LIT	LSTM-RNN combined with TSR for noise reduction	Model effectiveness might vary with different defect types and depths	Defect depth data from CFRP sheets	$R^{2}$ (0.78–93)
Kang et al. [198]	Addressing nonlinear issues in CDPRs with polymer cables	Hybrid RNN (H-RNN) combining LSTM and basic RNN for position error prediction	Model complexity may limit its application to simpler systems	Position error data from CDPRs	N/A
Lin et al. [201]	Real-time prediction of HFR in PEMFCs	LSTM model using current and past sensor data	Model effectiveness may decrease with changes in PEMFC operational conditions	HFR data from PEMFCs	MAPE (2.82%)
Lorenzo et al. [202]	Classifying plastics using hyperspectral images	1D CNN and SVM+RBF models	Requires extensive hyperspectral data; may be limited to specific plastic types	Hyperspectral image data from plastics	Accuracy (99.41%)
Choi et al. [203]	Enhancing mechanical stability and motion detection in PBU/AgNW/PBU sensors	1D CNN and LSTM models for motion detection	Limited testing in real-world applications; further validation required	Motion detection data from sensors	Accuracy (98%)

## Data Availability

Not applicable.

## Abbreviations

The following abbreviations are used in this manuscript:

Machine learning (ML)	A field of artificial intelligence focused on developing algorithms that enable computers to learn from data.
Long Short-Term Memory (LSTM)	A type of recurrent neural network capable of remembering long-term dependencies in data.
Artificial neural network (ANN)	A mathematical model inspired by the neural network of the brain, used for data processing and decision making.
Charge-Coupled Device (CCD)	An electronic device used for capturing images in digital cameras and telescopes.
Facial recognition (Facial Recog.)	Technology for identifying or verifying a person’s identity based on their facial image.
Object tracking (Obj. Tracking)	The process of following the movement of an object in a sequence of images or video.
Chemical sensor arrays (Chem. Sensor Arrays)	A system of multiple sensors used for detecting and analyzing chemical substances.
Temperature response (Temp. Resp.)	The change in system parameters in response to a change in temperature.
Neural architecture (Neural Arch.)	The structure and organization of a neural network.
Chemical awareness (Chem. Awareness)	The ability of a system to detect and identify chemical substances.
Dynamic environments (Dyn. Envs.)	Changing or unstable conditions in which a system operates.
Carbon black	A black carbon powder used as a filler in rubber and plastics.
Organic polymers (Org. Polymers)	Polymers made of carbon compounds, widely used in various fields.
Poly(4-vinyl phenol) (P(4-vinyl phenol))	A polymer used in electronics manufacturing and coatings.
Poly(styrene-co-allyl alcohol) (P(styrene-co-allyl alcohol))	A copolymer used in plastics and coatings.
Poly(ethylene oxide) (P(ethylene oxide))	A polymer used in medicine, cosmetics, and the textile industry.
Classification tasks (Class. Tasks)	Tasks related to categorizing data into classes or groups.
Traffic sign recognition (Traffic Sign Recog.)	Technology for recognizing traffic signs for use in automated driving systems.
Olfactory signal classification (Olf. Signal Class.)	The process of classifying smells based on signals obtained from olfactory sensors.
Temperature dynamics (Temp. Dyn.)	The study of temperature change in a system over time.
Olfactory sensing systems (Olf. Sensing Sys.)	Systems that use sensors to detect and analyze odors.
Extended Kalman Filter (EKF)	A filtering algorithm used for state estimation in nonlinear dynamic systems.
State-of-charge estimation (SOC Est.)	The estimation of a battery’s charge level based on measured data.
Lithium polymer batteries (Li-poly Batteries)	A type of battery with a polymer electrolyte, known for high energy density.
Battery management system (BMS)	A system that monitors and optimizes battery performance.
Carbon fiber-reinforced polymer (CFRP)	A composite material made from carbon fiber, known for high strength and low weight.
Laser infrared thermography (Laser IR Thermography)	A diagnostic method using infrared laser for temperature measurement in materials.
Defect depth assessment (Def. Depth Assess.)	Determining the depth of defects in materials or structures.
Traffic sign recognition (TSR)	The process of automatically recognizing traffic signs.
Generative DL (Generative DL)	A branch of DL focused on generating new data based on a trained model.
Ultraviolet–visible spectra (UV-vis Spectra)	Absorption and reflection spectra in the ultraviolet and visible range, used for substance analysis.
Coarse-grained models	Models that simplify complex systems while retaining essential characteristics.
Cable-driven robots	Robots controlled by a system of cables or wires.
Nonlinear characteristics (Nonlinear Char.)	Properties of a system or material that do not follow linear laws.
Real-time control	The control of processes in real time.
Hierarchical recurrent neural network (H-RNN)	A variant of recurrent neural network with a hierarchical structure.
Composite damage (Comp. Damage)	Damage to composite materials under various factors.
Finite element model (FE Model)	A numerical model used for solving problems in solid mechanics using the finite element method.
Twisted-coiled actuators	Actuators made of twisted and coiled fibers that change shape in response to temperature or electrical current.
Model Predictive Control (Model Predict. Control)	A control algorithm that uses predictive models to optimize system performance.
Organic photovoltaic materials (OPV Materials)	Organic materials used for making solar cells.
Simplified Molecular Input Line Entry System Fingerprints (SMILES Fingerprints)	A string-based encoding of chemical structures used for molecular analysis and comparison.
Polymer repeat units	The basic structural elements that make up polymers.
Glass fiber-reinforced polymer (GFRP)	A composite material reinforced with glass fiber, used in construction and engineering.
Terahertz time-domain spectroscopy (Terahertz Time-Domain Spec.)	A method for studying materials using terahertz radiation.
Dielectric electroactive polymer actuators (DEAP Actuators)	Actuators based on dielectric electroactive polymers that change shape when an electric field is applied.
Hysteresis	A phenomenon where the state of a system depends on its previous states despite identical current conditions.
Empirical mode decomposition (EMD)	A method for signal analysis that decomposes signals into component frequencies.
Battery state-of-charge estimation (Battery SOC Est.)	Estimation of a battery’s state of charge.
Plastic recycling	The process of recycling plastics for reuse.
Hyperspectral imaging	A method of acquiring and analyzing images that include spectral information across a wide range of wavelengths.
Polymer insulation resistance (Polymer Ins. Resist.)	A polymer’s ability to resist electrical leakage.
Melt index	A measure of the flow rate of a polymer when melted under specific conditions.
Polymerization processes	Chemical processes in which monomers combine to form polymers.
Acoustic behavior	The characteristics of a system related to the generation, transmission, and absorption of sound waves.
Muffler design	The design and construction of mufflers to reduce noise.
Soft sensor	A software tool for estimating system parameters based on indirect measurements.
Dielectric electroactive polymer actuation (DEAP Act.)	The actuation process of a device based on dielectric electroactive polymers.
Proportional–integral–derivative controller (PID Controller)	A control algorithm using three components: proportional, integral, and derivative.
Ethyl acetate solution (Ethyl Acetate Sol.)	A solution of ethyl acetate, used in various chemical processes.
Hybrid sensor	A sensor that combines multiple technologies to enhance accuracy and functionality.
Motor tics recognition (Motor Tics Recog.)	A system for recognizing motor tics in individuals based on movement analysis.
Polymethyl methacrylate (PMMA)	A transparent thermoplastic widely used in construction and medicine.
Heat transfer	The process of transferring heat from one object to another.
High-temperature proton exchange membrane fuel cell (High-Temp PEMFC)	A fuel cell with a proton exchange membrane operating at high temperatures.
Hydrogen starvation (H2 Starvation)	A condition where a fuel cell receives insufficient hydrogen.
Nafion membranes	Proton-conducting polymer membranes used in fuel cells.
Flooding and drying	Phenomena occurring in fuel cells due to excess moisture or drying of the membrane.
Tool wear prediction (Tool Wear Pred.)	Predicting tool wear using data analysis and modeling.
Polybutadiene-urethane	A polymer used as an elastomer or coating.
Motion detection	Technology for detecting movement in space using sensors or cameras.
Knot identification (Knot Ident.)	The process of recognizing knots in a rope or cord.
Structural health monitoring (SHM)	Monitoring the condition of structures to detect defects or damage.
Lamb wave	A type of elastic wave that propagates in solid materials and is used for diagnostics.
Variational mode decomposition (VMD)	A signal decomposition method for analyzing various modes of a signal.
